# Signatures of Selection in Admixed Dairy Cattle in Tanzania

**DOI:** 10.3389/fgene.2018.00607

**Published:** 2018-12-19

**Authors:** Evans Kiptoo Cheruiyot, Rawlynce Cheruiyot Bett, Joshua Oluoch Amimo, Yi Zhang, Raphael Mrode, Fidalis D. N. Mujibi

**Affiliations:** ^1^Department of Animal Production, College of Agriculture and Veterinary Sciences, University of Nairobi, Nairobi, Kenya; ^2^USOMI Limited, Nairobi, Kenya; ^3^College of Animal Science and Technology, China Agricultural University, Beijing, China; ^4^International Livestock Research Institute, Nairobi, Kenya; ^5^Scotland’s Rural College, Edinburgh, United Kingdom; ^6^Nelson Mandela African Institute of Science and Technology, Arusha, Tanzania

**Keywords:** selection signatures, crossbred cattle, admixture, iHS, XP-EHH, pcadapt, SNP

## Abstract

Multiple studies have investigated selection signatures in domestic cattle and other species. However, there is a dearth of information about the response to selection in genomes of highly admixed crossbred cattle in relation to production and adaptation to tropical environments. In this study, we evaluated 839 admixed crossbred cows sampled from two major dairy regions in Tanzania namely Rungwe and Lushoto districts, in order to understand their genetic architecture and detect genomic regions showing preferential selection. Animals were genotyped at 150,000 SNP loci using the Geneseek Genomic Profiler (GGP) High Density (HD) SNP array. Population structure analysis showed a large within-population genetic diversity in the study animals with a high degree of variation in admixture ranging between 7 and 100% taurine genes (dairyness) of mostly Holstein and Friesian ancestry. We explored evidence of selection signatures using three statistical methods (iHS, XP-EHH, and pcadapt). Selection signature analysis identified 108 candidate selection regions in the study population. Annotation of these regions yielded interesting genes potentially under strong positive selection including ABCG2, ABCC2, XKR4, LYN, TGS1, TOX, HERC6, KIT, PLAG1, CHCHD7, NCAPG, and LCORL that are involved in multiple biological pathways underlying production and adaptation processes. Several candidate selection regions showed an excess of African taurine ancestral allele dosage. Our results provide further useful insight into potential selective sweeps in the genome of admixed cattle with possible adaptive and productive importance. Further investigations will be necessary to better characterize these candidate regions with respect to their functional significance to tropical adaptations for dairy cattle.

## Introduction

Livestock genomes have undoubtedly undergone significant changes following domestication about 10,000 years ago (Loftus et al., 1994) and subsequent breed formation through natural and artificial selection. Identification of selection footprints occasioned by such domestication events has been a subject of intense research in recent years motivated mainly by the desire to understand the molecular mechanisms involved in the adaptation events as well as identify genomic regions associated with phenotypic variation (Andersson and Georges, 2004). The increasing availability of single nucleotide polymorphism (SNP) data and unprecedented reduction in genotyping cost offers a unique opportunity for detailed assessment of genetic diversity and localizing selective sweeps at greater resolution. This study sought to understand the genomic structure and selection signature profile of admixed Tanzanian crossbred dairy cattle that have been subjected to tropical conditions.

In developing countries, crossbreeding is considered an effective management strategy that allows exploitation of a combination of the high adaptability to environmental conditions, resistance to diseases and overall hardiness possessed by local indigenous breeds alongside the relatively high productive potential of exotic breeds (Mbole-Kariuki et al., 2014; Leroy et al., 2016). Consequently, this breeding strategy has been widely practiced in tropical countries. In Tanzania, crossbred dairy cattle are mainly the product of crossbreeding of local East African Shorthorn Zebu (EASZ) and imported exotic dairy breeds (mainly Holstein, Friesian and Ayrshire) that began during the colonial period and further promoted by the government during the 1980s (Mwenya, 1993). These animals are mainly kept in smallholder farms and have been subjected to natural and non-systematic artificial selection for economic traits such as milk yield, growth rate and reproduction efficiency (Bebe et al., 2003). Additionally, exposition to multiple environmental stresses such as infectious diseases, high ambient temperatures, and poor feed is the norm (Swai et al., 2010). Given such a landscape, it is likely that there are footprints of selection linked to adaptation and productivity in challenging tropical production conditions. Due to the significance of dairy farming to smallholder farmers in East Africa, it is critical that the determinants of adaptation and production be well understood. This is particularly important considering the need to improve the low production of these cattle – the average daily milk yield per cow for crossbred cattle in Tanzania is 5.8 l in smallholder systems (Makoni et al., 2014).

Various statistical methods have been developed to detect footprints of selection based on neutral evolutionary theory (Biswas and Akey, 2006). These methods can be broadly divided into three main classes: (a) measures based on the allele frequency (e.g., *Tajima’s* D; *Fu* and *Li* test) (b) within population measures based on extended haplotype homozygosity (e.g., iHS) and (c) measures based on differentiation between and within species (e.g., XP-EHH, Fst or related statistics and principal component analysis) as reviewed by Vitti et al. (2013). Application of these methods in livestock species has revealed widespread signatures of selection in the genome of domestic cattle linked to environmental adaptation and production traits (Randhawa et al., 2016). However, few studies on admixed crossbred cattle have demonstrated evidence of recent positive selection related to adaptation in East Africa (Kim and Rothschild, 2014). Similar studies have also demonstrated evidence of selective sweep in several admixed cattle including Swiss Fleckvieh cattle (Khayatzadeh et al., 2016). Creole cattle (Gautier and Naves, 2011), Borgou and Baoule breeds (Flori et al., 2014; Smetko et al., 2015), East African Shorthorn Zebu (Bahbahani et al., 2015) and recently in admixed Kenana and Butana zebu (Bahbahani et al., 2018). Analytical procedures for detection of selection sweeps continue to emerge. Methods that are somewhat robust to admixture have been developed, including pcadapt (Luu et al., 2017), FLK (Bonhomme et al., 2010), hapFLK (Fariello et al., 2013). More recently, a local score approach (an extension of FLK) which clusters selection signals based on p-values while accounting for linkage disequilibrium (LD) has been developed (Fariello et al., 2017). Although these new approaches have improved detection and resolution of selection regions, distinguishing between true selection signals and those that merely arise from drift remains a challenging task, particularly for recently admixed breeds (Akey et al., 2002; Teshima et al., 2006; Pierron et al., 2018). A different approach that has been proposed as ideal for exploring selection signatures in the presence of admixture, is the Efficient Local Ancestry Inference (ELAI) algorithm (Guan, 2014). This method infers local ancestry and aims at detecting regions where one genetic component deviates from average genome-wide ancestry (Guan, 2014; Zhou et al., 2016).

In this study, we applied three complementary approaches: iHS, XP-EHH, and pcadapt to explore selection signatures in the autosomal genome of Tanzanian crossbred dairy cows, with a view of understanding the possible determinants of adaptation and production and to relate these signals to adaptation and productivity traits. In addition, we examined the local ancestral allele dosage of the putative selection regions using ELI algorithm (Guan, 2014) to understand ancestral origins.

## Methodology

### Ethics Statement

This study was undertaken according to the International Livestock Research Institute (ILRI) Institutional Animal Care and Use Committee (IACUC) guidelines, with approval reference number 2014.35. Animals were handled by experienced animal health professionals during blood and hair sampling to minimize discomfort and injury. Prior to sample collection, meetings were held with farmers to explain the purpose of the study and obtain informed consent.

### Animal Resources

Samples were collected from two districts of Tanzania, namely Rungwe and Lushoto located in the Southern and Northern Highlands, respectively. These study sites were chosen based on the availability of a wide range of breeds, the population density of improved dairy cattle, the presence of complimentary dairy projects led by ILRI under the ‘Maziwa Zaidi’ program, and the sites having been identified as emerging high dairy potential regions. Both districts have similar agro-ecological climates owing to their locations in high altitude zones with mixed crop-livestock farming being one of the major economic activity. However, unlike Lushoto district, there is greater emphasis on dairy farming in Rungwe with the majority of dairy animals being fed under zero grazing conditions (Mwakaje, 2008). The history of dairy farming is scanty in Lushoto but more established in Rungwe supported previously by international breed organizations dating back to 1970s (Mwakaje, 2008).

Blood samples were collected by venipuncture using approved procedures that avoid unnecessary pain and suffering. The procedure was undertaken by qualified veterinarians. Hair samples were collected from the tail switch of the animals, taking care to avoid fecal contamination following the protocol described by the Animal Genetics Laboratory (2013). A total of 839 cows were sampled from smallholder dairy farms consisting of 490 samples from the Rungwe district and 349 samples from the Lushoto district.

### Reference Dataset

A panel of genotypes from commercial international taurine dairy breeds was used as a reference for breed composition assignment. These included Friesian (28 samples), Holstein (63), Norwegian Red (17), Jersey (36), and Guernsey (21) breeds. To capture genetic signatures representative of African cattle, an African taurine breed (N’Dama (24)) and two indicine breeds, the East African Shorthorn Zebu (EASZ) (50) and Gir (30) were also included in the analysis.

### Genotyping and Quality Control

Samples were genotyped at Geneseek (Neogen Corporation, Lincoln, NE, United States) using the Geneseek Genomic Profiler (GGP) High Density (HD) SNP array consisting of 150,000 SNPs, while SNPs for the reference breeds had been genotyped with the Illumina HD Bovine (777K SNPs) array. The SNPs in GGP array are optimized for use in dairy cattle having the most informative SNPs from Illumina Bovine 50 and 770 k chips and additional variants known to have a large effect on disease susceptibility and performance. Before analysis, the study genotypes were merged with the reference genotypes using PLINK (Purcell et al., 2007), resulting in 134,295 overlapping SNPs. Next, genotype data quality control and checks were performed as described in Cheruiyot et al. (2018) by removing SNPs with less than 90% call rate, less than 5% minor allele frequency (MAF), and samples with more than 10% missing genotypes. Additional removal of SNPs not mapped to any chromosome left a total of 111,836 SNPs for analysis.

### Minor Allele Frequency, Inbreeding and Heterozygosity Estimates

Minor allele frequencies (MAF) were estimated using PLINK (Purcell et al., 2007). The distribution of MAF in each subpopulation (i.e., European taurine, African taurine, Indicine breeds, and Tanzanian crossbred cattle) was represented as the proportion of all the SNPs used in the analysis and subsequently grouped into five classes as follows: [0.0,0.1], [0.1,0.2], [0.2,0.3], [0.3,0.4], [0.4,0.5]. The results were plotted for comparison between subpopulations using R v. 3.4.4 (R Core Team, 2018).

The observed heterozygosity estimates for each population were calculated from observed genotype frequencies obtained from PLINK (Purcell et al., 2007) as follows: (N - O)/N (where N is the number of non-missing genotypes and O is the number of observed homozygous genotypes for a given individual).

The inbreeding coefficient (F) was calculated using PLINK based on the observed versus expected number of homozygous genotypes as follows:

$$F=f_{\text{i}}+(1-f_{\text{i}})(p^{2}+q^{2})$$

where *f_i_* is the probability of individual *i* being homozygous by descent, (1 - *f_i_*) is the probability that individual *i* is homozygous by chance for a specific SNP with known allele frequencies *p* and *q* (Purcell et al., 2007). Before analysis, SNPs were pruned to obtain markers in approximate linkage equilibrium. This was done in PLINK program using the –indep-pairwise (50 5 0.3) option. The pruning proceeded by calculating LD for 50 marker sliding windows, with a new window obtained by shifting 5 markers along the length of the chromosome. Marker pruning was effected if LD between a pair of markers was 0.3 or above. Consequently, 62,475 markers were removed leaving a total of 67,496 markers that were used for the inbreeding analysis.

### Admixture and Principal Component Analysis

To accurately describe the population structure of the crossbred cattle population, we used PC-AiR method to perform principal component analysis (PCA) using GENESIS package (Conomos et al., 2015) in R v. 3.4.4 (R Core Team, 2018). PCA results were then visualized using the GENESIS package (Buchmann and Hazelhurst, 2014).

The unsupervised model-based clustering method implemented by the program ADMIXTURE v. 1.3.0 (Alexander et al., 2009) was used to estimate the breed composition of individual animals using 111,836 markers. The analysis was run with K (number of distinct breeds) ranging from 2 to 9 to reflect the genetic background of the cattle under study, starting with the basic cross (indicine and taurine cross) until the total number of the populations in the analysis, given the 8 reference breeds. Ten-fold cross-validation (CV = 10) was specified, with the error profile obtained thereafter used to explore the most probable number of clusters (K), as described by Alexander et al. (2009). Graphical display of the admixture output was done using the Genesis package (Buchmann and Hazelhurst, 2014) in R v. 3.4.4 (R Core Team, 2018).

### Identification of Selection Signatures

Signatures of selection analyses were performed using 111,836 SNPs that remained after quality control and checks. Three complementary statistical methods were used to detect putative selection signatures. Two tests, integrated haplotype score (iHS) (Voight et al., 2006) and the cross-population extended haplotype-based homozygosity score test (XP-EHH) (Sabeti et al., 2007) were based on LD patterns while an outlier test pcadapt (Luu et al., 2017) was based on allele frequency differentiation. Haplotypes for iHS and XP-EHH analyses were derived using *fastPHASE* (Scheet and Stephens, 2006) by applying the criteria K20, T10 C25, where K is the number of clusters; T and C are the number of starts and number of iterations of EM algorithm, respectively (Scheet and Stephens, 2006). Additionally, iHS analysis was performed using the *rehh* package (Gautier and Vitalis, 2012) in R v. 3.4.4. The iHS statistic is a within population statistic which measures the amount of extended haplotype homozygosity (EHH) for a given SNP along the ancestral allele relative to the derived allele. In this study, the ancestral alleles required for the computation of iHS were inferred as the most common alleles in the entire dataset as described by Bahbahani et al. (2015). In order to allow better visualization and comparison of selection signals, | iHS| scores were transformed into - log 10 [1 - 2| Φ iHS - 0.5|] in which Φ iHS is the cumulative Gaussian distribution function of iHS. *P*-values were calculated as described in Gautier and Naves (2011). We applied the method of Storey and Tibshirani (2003) to control false positives at a false discovery rate (FDR) threshold of 1% which corresponded to a *p*-value of < 0.0001.

Using EASZ as a reference population, we calculated XP-EHH scores for Tanzanian crossbred cattle. XP-EHH compares the extended haplotype homozygosity between two populations at each focal SNP and allows detection of recent selection events, in which haplotypes have almost or fully risen to fixation (Sabeti et al., 2007). As in iHS, the XP-EHH scores were standardized to a distribution with zero mean and unit variance to enable better visualization and interpretation of regions under selection. Additionally, *p*-values were calculated as described in Gautier and Naves (2011) and FDR performed following Storey and Tibshirani (2003) with the threshold set at 1%.

To identify outlier loci, we performed analysis using pcadapt package which implements PCA (Luu et al., 2017). pcadapt is robust to admixture and does not assume prior knowledge of population structure. The analysis was performed on a combined dataset of Tanzanian crossbred population, EASZ and N’Dama. As recommended, we applied Cattell’s graphical rule (Cattell, 1966) to decide on the number of the principal components to retain. The test statistic for pcadapt is the Mahalanobis distance (D) which is calculated from a vector of z-scores obtained by regressing each SNP with K principal components, defined as:

$$D_{j}^{2}={\left(z_{j}-\bar{z}\right)}^{T}\sum^{-1}(z_{j}-\bar{z})$$

where, Σ is the (K × K) covariance matrix of the *z*-scores and 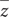 is the vector of the K *z*-score means (Luu et al., 2017). The *p*-values are obtained from transforming Mahalanobis distance (D) based on the chi-square distribution. To identify outlier SNPs, we applied the approach of Storey and Tibshirani (2003) based on FDR at 1%.

### Local Ancestry Estimation of Candidate Selection Regions

We inferred local ancestry using Efficient Local Ancestry Inference (ELAI) algorithm (Guan, 2014) in order to understand the ancestral origins of the major selection regions in the Tanzanian crossbred population. Before analysis and to minimize computational resources, we filtered related individuals using KING program v. 2.1.5 by specifying the –unrelated option (Manichaikul et al., 2010). A total of 324 unrelated animals remained for ancestry inference (146 and 178 animals for Lushoto and Rungwe, respectively).

We run ELI assuming three source populations: (a) European taurine based on a dataset that combined all the Commercial European dairy breeds used as reference (b) African taurine (represented by N’Dama) and (c) indicine breed (represented by EASZ). Analysis was performed only for the chromosomes harboring strong candidate selection regions detected by iHS or pcadapt approaches (i.e., BTA5, BTA6, BTA7, and BTA14). Data quality checks included removal of SNPs with MAF < 0.05 as well as SNP with missingness > 0.05. The upper and lower layer clusters were set as 3 and 15, respectively. ELI requires specification of the number of admixing generations. Thus, we specified 10 admixture generations, assuming a 5-year generational interval. These generations correspond to the recent history of crossbreeding in Tanzania supported by the reports indicating that crossbreeding in Tanzania became prominent 1960s when smallholder dairy farming began in earnest post-independence (Mwenya, 1993; Nell et al., 2014). As recommended, we run the analysis for 10 independent EM runs of 20 steps each and averaged results for all the 324 individuals at each locus. We then performed Grubb’s test for outlier using outlier package in (Komsta, 2011) R v. 3.4.4.

### Annotation of Significant Regions

We annotated genomic regions under significant selection pressure using the Ensemble Biomart tool^1^ based on UMD v3.1 bovine genome assembly. To limit possible false positives, we designated a region as candidate selective sweep if it passed the 1% FDR threshold and contained at least two SNPs separated by not more that than 500 kb. This was done by first identifying any two SNPs which are not separated by 500 kb and then cumulating consecutive SNPs on both sides until the last SNP is separated by >500 kb. The window chosen was informed by previous evidence that the LD in cattle do not exceed 500 kb (McKay et al., 2007). For each analysis, genes within a region spanning 100 kb upstream and downstream of the candidate selection regions were annotated.

## Results

### Sample Description and Characteristics of the Marker Panel Used

Table 1 provides summary statistics of the samples used in the study whereas Supplementary Table S1 provides details about the maker set used in the analysis. The 111,836 SNPs that remained after quality control and checks covered 2516.25 Mb with an average distance of 22.67 kb between adjacent SNPs. The mean chromosomal length ranged between 42.8 Mb on BTA 25–158.86 Mb on BTA 1. The mean length of adjacent SNPs per chromosome ranged between 18.67 to 23.89 kb on BTA 14 and BTA 29, respectively. The LD across the genome averaged 0.41.

**Table 1:** Sample description for Tanzanian crossbred cattle and reference breeds.

Breed/subpopulation	Abbreviations	Type	*N*^1^	Observed heterozygosity (±SD^2^)	Inbreeding coefficient (±SD^2^)
Lushoto	-	Crossbred	485	0.384 ± 0.02	0.033 ± 0.03
Rungwe	-	Crossbred	346	0.389 ± 0.014	0.02 ± 0.037
Holstein	HO	EUT^3^	63	0.368 ± 0.01	0.073 ± 0.03
Friesian	FR	EUT	28	0.362 ± 0.01	0.089 ± 0.03
Norwegian Red	NR	EUT	17	0.356 ± 0.00	0.104 ± 0.02
Jersey	JE	EUT	36	0.308 ± 0.01	0.225 ± 0.04
Guernsey	GN	EUT	21	0.312 ± 0.01	0.213 ± 0.04
N’Dama	ND	AUT^4^	24	0.245 ± 0.01	0.384 ± 0.02
East African Shorthorn Zebu	EASZ	Indicine	50	0.284 ± 0.01	0.285 ± 0.04
Gir	GI	Indicine	30	0.206 ± 0.00	0.481 ± 0.02

*^*1*^Number of samples; ^*2*^Standard deviation; ^*3*^European taurine; ^*4*^African taurine.*

### Genetic Diversity

**FIGURE 1:**
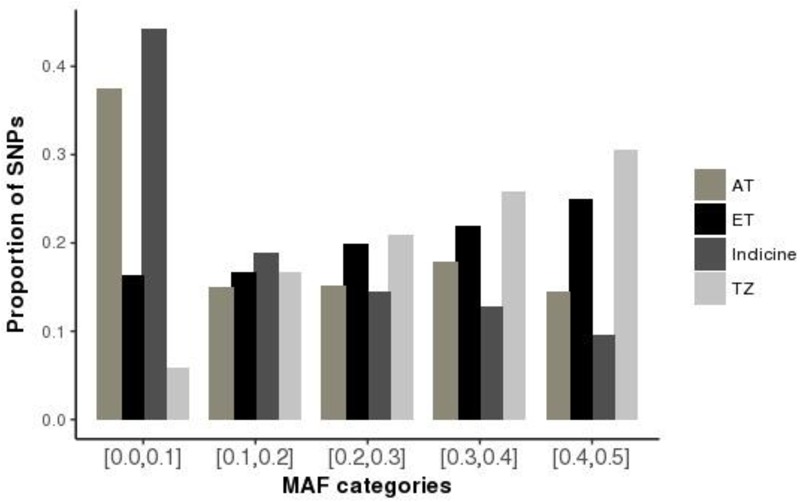
Distribution of minor allele frequencies (MAF) for the Tanzanian crossbred cattle and reference breeds. Subpopulations are indicated as AT, African Taurine; ET, European Taurine; Indicine and Tanzanian crossbred cattle (TZ), respectively. SNPs were SNPs were binned into 5 categories based on the MAF; [0, 0.1], [0.1, 0.2], [0.2, 0.3], [0.3, 0.4], and [0.4, 0.5].

The distributions of average minor allele frequencies for all populations under study (African taurine, Indicine, and Tanzanian crossbred cattle) are shown in Figure 1. Indicine (East African Shorthorn Zebu and Gir) and African taurine (N’Dama) breeds had the highest proportion of SNPs with the low MAF category ([0.0, 0.1]) compared to European taurine (ET) breeds. The Tanzanian crossbred cattle had a relatively high proportion of SNPs with high MAF (mostly [0.3, 0.4] and [0.4, 0.5]).

The observed heterozygosity estimates for the study populations are provided in Table 1 and illustrated in Supplementary Figure S1. The average heterozygosity estimates were highest for the crossbred cattle (38.4 ± 2% and 38 ± 1% Lushoto and Rungwe, respectively), and lowest for indicine breeds (28.4 ± 2% for East African Shorthorn Zebu and 21 ± 1% for Gir) as well as African taurine breed (N’Dama) at 24.5 ± 1%. Heterozygosity estimates for European taurine breeds ranged between 30.8 ± 1.6% and 36.8 ± 1.4% for Jersey and Holstein breeds, respectively.

The study populations showed low detectable levels of inbreeding. The inbreeding coefficient estimates were slightly higher for cattle in Lushoto (3.3 ± 3.6%) compared to Rungwe (2.0 ± 3.7%), as shown in Table 1. However, these values were not significantly different from zero (*p* < 0.001).

### Principal Component Analysis

**FIGURE 2:**
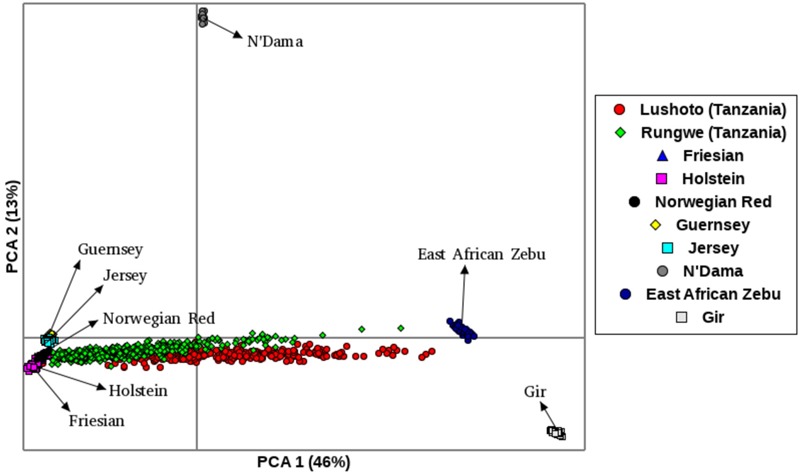
Principal Component Analysis (PCA) plot showing clustering of Tanzanian crossbred cattle and reference breeds. Each point represents an individual animal colored as per the breed.

The first principal component (PC1), accounted for 46% of the total variation and separated European taurine breeds from non-European breeds as shown in Figure 2. The second component (PC2) accounted for 13% of the total variation and separated African breeds (N’Dama, EASZ) from non-African breeds. Tanzanian samples dispersed along the PC2 coordinate clustering intermediate between East African Shorthorn Zebu (EASZ) and Friesian breeds. The first (PC1) and third component (PC3) (Supplementary Figure S2) explained 46 and 5%, respectively of the total variation and separated breeds based on their geographic origin: the Channel Islands breeds: Jersey (JE) and Guernsey (GN) vs. the Northern European taurine breeds: Holstein (HO), Norwegian Red (NR) and Friesian (Gautier et al., 2010). Additionally, PC1 and PC3 show a clear definite dispersion of Tanzanian crossbred cattle toward Northern European taurine breeds [Holstein (HO), Norwegian Red (NR) and Friesian (FR)] (Supplementary Figure S2). Moreover, a larger proportion of the animals from Rungwe (green colored) clustered closer to the European taurine breeds compared to those from Lushoto (red colored), which were more dispersed toward the EASZ.

### Admixture Analysis

**FIGURE 3:**
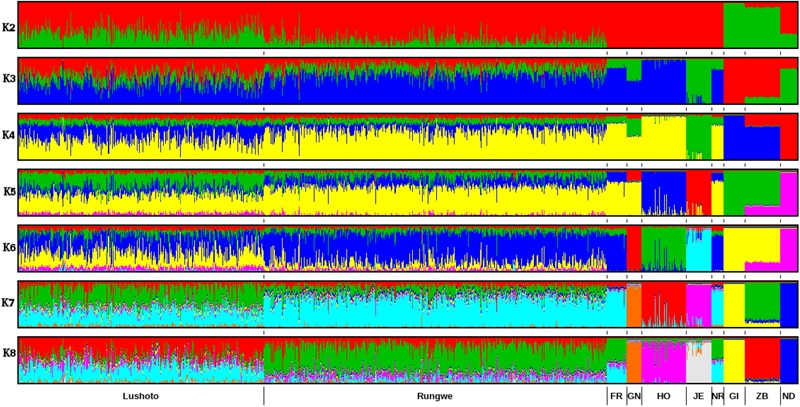
ADMIXTURE bar plot showing breed proportions at assumed ancestry (cluster) K = 2–8. Short vertical lines at the bottom of each horizontal bar delimit individuals of different populations. Tanzanian crossbred cattle populations are divided according to the sampling locations (Lushoto and Rungwe) while reference breeds are labeled as Friesian (FR), Holstein (HO), Guernsey (GN),Jersey (JE), Norwegian Red (NR), Gir (GI), East African Shorthorn Zebu (ZB), and N’Dama (ND).

ADMIXTURE results for *K* = 2 to *K* = 8 are presented in Figure 3 while the cross-validation (CV) error plot is presented in (Supplementary Figure S3). CV error is used to predict the most appropriate value for K (the optimal number of populations in the dataset) (Alexander et al., 2009). In this study, the CV errors continued to decrease as *K* increased in value in the combined dataset of reference and Tanzania data, hence no clear indication of the appropriate *K* for our population was obtained using this statistic. Based on visual inspection of the admixture plot, scrutiny of the separate CV error plots and the PCA plots, *K* = 7 represented the most appropriate population number for the dataset. Importantly, increasing *K* above 7 did not reveal any detectable population substructure and the breed clusters remained the same.

Based on results obtained with *K* = 7, most animals were crosses of Holstein, Friesian and Red breeds (which formed a single cluster in the ADMIXTURE plot), which contributed on average 50% of the total genes in the crossbred animals. The predicted absolute exotic breed gene content in the crossbred cattle ranged from 7 to 100%. Rungwe cattle had significantly (*p* < 0.001) higher levels of taurine admixture (mean 78.3 ± 13%) compared to those in Lushoto (mean 56.4 ± 16%).

### Selection Signatures Based on iHS

**FIGURE 4:**
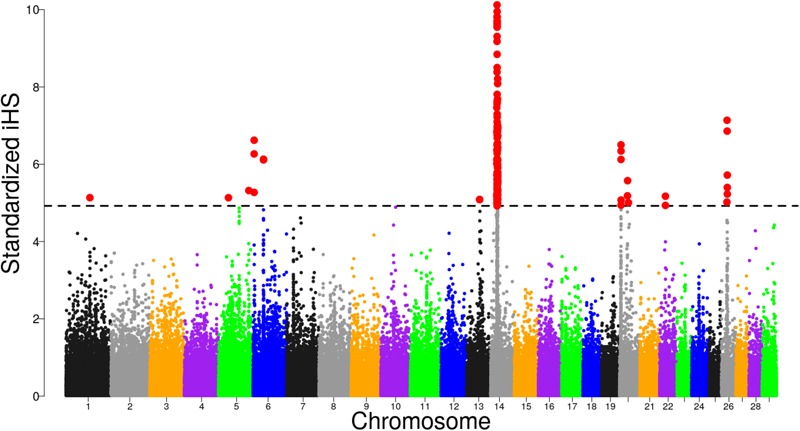
Distribution of standardized iHS scores in the Tanzanian crossbred cattle versus EASZ comparison. The dashed line corresponds to the false discovery rate (FDR) at 1% threshold.

Focusing on Tanzanian crossbred cattle, several significant regions were detected after FDR adjustment at 1% on BTA, 1, 5, 6, 13, 14, 20, 22, and 26 (Figure 4). Of these regions, only selective sweeps on BTA 6, 14, 20, 22, and 26 passed the clustering criteria (see materials and methods) for designating candidates for strong selection. Notably, a strong selective sweep was observed on BTA 14 at 23.28 – 26.99 Mb.

Previous studies have demonstrated strong evidence for the presence of a causal mutation BovineHD1400007259 (rs109815800) intronic to PLAG1 gene at position 25015640 (Karim et al., 2011; Boitard et al., 2016; Bouwman et al., 2018). This position is within the iHS sweep region. To understand if the strong signal observed in the sweep region could be associated with the QTN, we examined allele frequency patterns in Tanzanian crossbred cattle and compared with reference breeds. We found that the rs109815800 SNP is fixed or almost fixed in European taurine and N’Dama cattle but is segregating at intermediate frequencies in the Tanzanian crossbred cattle (Supplementary Figure S4). Examining the haplotype diversity within the BTA 14 sweep region showed that the Tanzanian crossbred cattle share a common haplotype background with N’Dama that is almost devoid in the Holstein and Friesian (Supplementary Figure S5).

### Selection Signatures Based on XP-EHH

**FIGURE 5:**
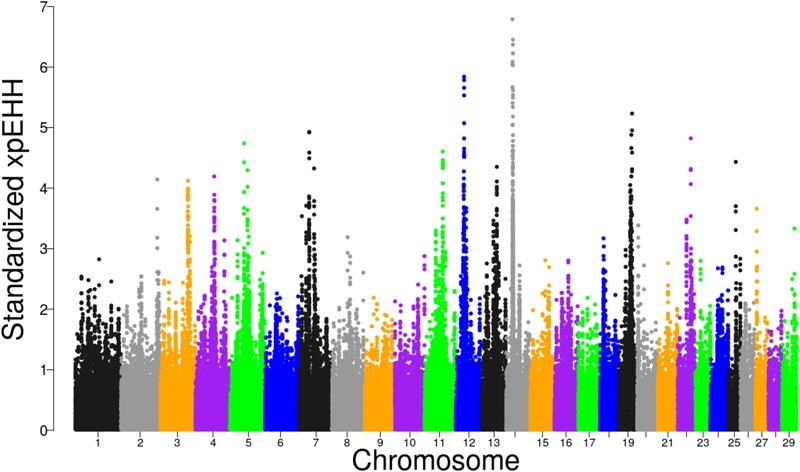
Distribution of standardized XP-EHH scores in the Tanzanian crossbred cattle versus the EASZ comparison.

The distribution of XP-EHH scores for Tanzanian crossbred cattle is shown in Figure 5. Using FDR threshold of 1% resulted in none of the selection sweeps being detected as significant. However, when the analysis was re-run with Rungwe and Lushoto populations separately, with FDR at 1%, a significant region on BTA 14 at 23.29 – 25.0 Mb was detected in Rungwe population (Supplementary Figure S6), similar as observed using iHS and pcadapt.

### Selection Signatures Based on Principal Component Analysis

**FIGURE 6:**
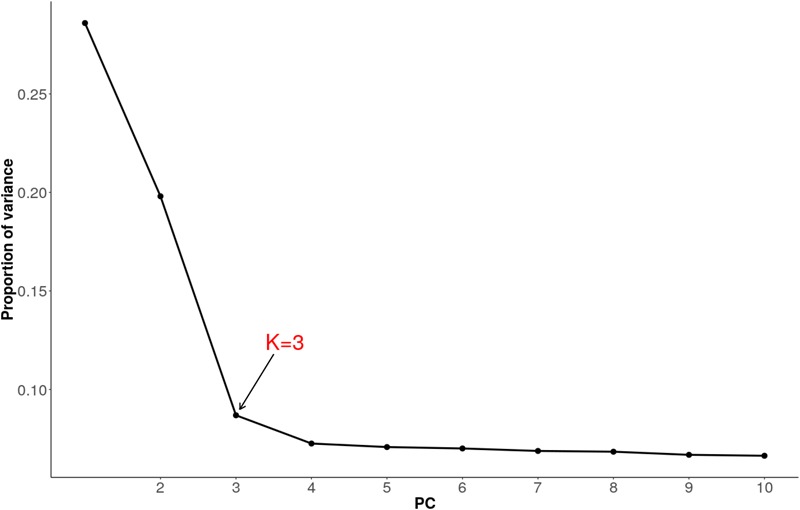
Proportion of variance explained by 10 principal components for combined dataset of Tanzanian crossbred cattle, East African Shorthorn Zebu (EASZ) and N’Dama.

We used pcadapt to detect outlier loci based on the PCA (Luu et al., 2017). As recommended, we applied Cattell’s graphical rule to choose the number of components to retain. The rule states that the last point before the curve flattens corresponds to the number of principal components which captures well the population structure. As such, heuristic inspection of the plot (Figure 6) clearly shows that three components (*K* = 3) should be retained.

**FIGURE 7:**
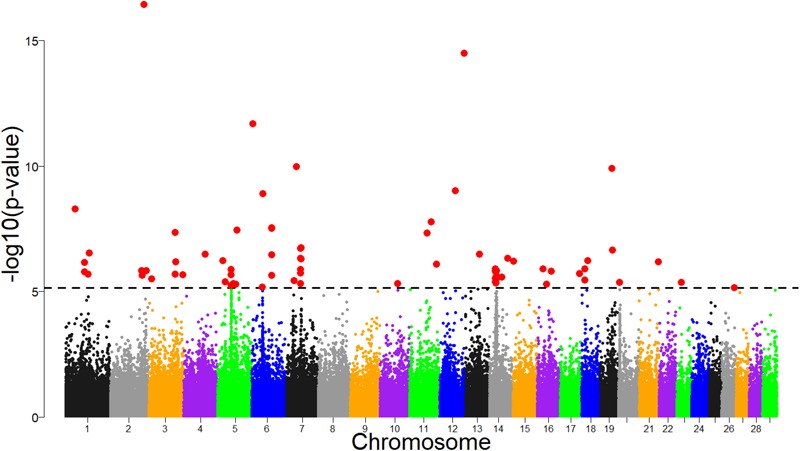
Manhattan plot of genomic selection regions detected using pcadapt for combined dataset of Tanzanian crossbred cattle, East African Shorthorn Zebu and N’Dama. The dashed line corresponds to the 1% false discovery rate (FDR) threshold.

Several significant regions were detected after FDR adjustment at 1% across the genome (Figure 7). Of these regions, candidate selection regions on BTA 6, 7, 14, 18 and 20 (Table 2) passed the clustering criteria (see Materials and Methods) for designating candidates for strong selection. The candidate regions ranged from 200 kb to 1.4 Mb in BTA 26 and BTA 7, respectively. Similarly, the largest number of SNPs (30) within a sweep region was found in BTA 7 at 51.2 – 52.4 Mb (Table 2). The genes for the significant selection regions which did not meet the clustering criterion are provided in Supplementary Table S3.

**Table 2:** Description of the candidate selective sweep regions detected using iHS and pcadapt analyses in Tanzanian crossbred cattle.

BTA	Selective sweep region (Mb)	No. of candidate genes	Top significant SNP	Genes mapping top SNPs	Detecting analysis	Maximum iHS/pcadapt statistic	*P*-value
BTA 6	37.82 – 37.89	6	BovineHD0600010455	PPM1K, HERC5, HERC6, ABCG2	iHS	4.94	7.7 × 10^-7^
	38.87 – 39.00	2	BovineHD0600010756	NCAPG, LCORL	pcadapt	41.19	1.1 × 10^-9^
	49.24 – 49.64	1	BovineHD0600001298	rRNA	iHS	5.01	5.4 × 10^-7^
	71.53 – 71.87	1	BovineHD0600019967	KIT	pcadapt	28.92	5.2 × 10^-7^
BTA 7	51.29 – 52.49	30	BovineHD0700015026	CTNNA1	pcadapt	24.60	4.5 × 10^-6^
BTA 13	45.87 – 46.18	0	BTB-00524844		iHS	4.48	8.2 × 10^-6^
BTA 14	23.49 – 23.86	5	BovineHD1400006897	RGS20, TCEA1, LYPLA1, MRPL15, POLR2K	pcadapt, iHS	29.06 (pcadapt)	4.8 × 10^-7^
	23.28 – 26.99	35	BTB-01532239	XKR4	iHS	-6.84	7.8 × 10^-12^
	26.47 – 26.68	2	BovineHD4100011326	TOX	pcadapt	30.82	2.0 × 10^-7^
BTA 18	12.74 – 12.88	2	BovineHD1800004310	FBXO31, C18H16orf95	pcadapt	25.29	3.2 × 10^-6^
BTA 20	44.14 – 46.25	2	BovineHD2000001436	DUSP1, ERGIC1	pcadapt	24.40	5.0 × 10^-6^
	28.25 – 28.95	1	BovineHD2000008382	PARP8	iHS	4.50	6.5 × 10^-6^
	57.43 – 57.79	1	BovineHD2000001806	NSG2	iHS	4.91	7.5 × 10^-7^
BTA 22	20.09 – 21.11	0	BovineHD2200006094		iHS	4.50	6.7 × 10^-5^
BTA 26	20.22 – 21.88	23	BovineHD2600005382	DNMBP	iHS	5.38	7.2 × 10^-8^

*The total number of genes refer to genes mapping 100 kb up/downstream of selective sweep regions. The genes mapping the most significant SNP(s) within the candidate regions are shown.*

### Overlapping Selection Regions

Based on the criteria of Voight et al. (2006), we define overlapping selection regions as those located above the cut-off threshold and in the same chromosomal location. Since no significant selection region was detected by XP-EHH at FDR of 1%, we considered overlapping candidate regions detected by iHS and pcadapt. Consequently, only one candidate sweep region on BTA 14 at 23.49 – 23.86 Mb was detected by both analyses (Table 2). It is important to point out that unlike iHS, the sweep for pcadapt was not contiguous from 23.28 to 26.99 Mb but was detected as two candidate sweeps based on the criterion (see materials and methods) (i.e., at 23.49 – 23.86 Mb and 26.47 – 26.68 Mb; Table 2).

### Local Ancestry of Candidate Selection Regions

We used Efficient Local Ancestry Inference (ELAI) algorithm (Guan, 2014) to investigate putative ancestral origins for the major selection regions detected in Tanzanian crossbred cattle. The global ancestry proportions estimated from ADMIXTURE (Alexander et al., 2009) for European taurine (ET), African taurine (AT) and indicine components were 70, 19, and 11%, respectively. The African taurine and indicine were significantly different in the study population (*t* = 4.53, df = 506.72, *p* < 0.00001).

**FIGURE 8:**
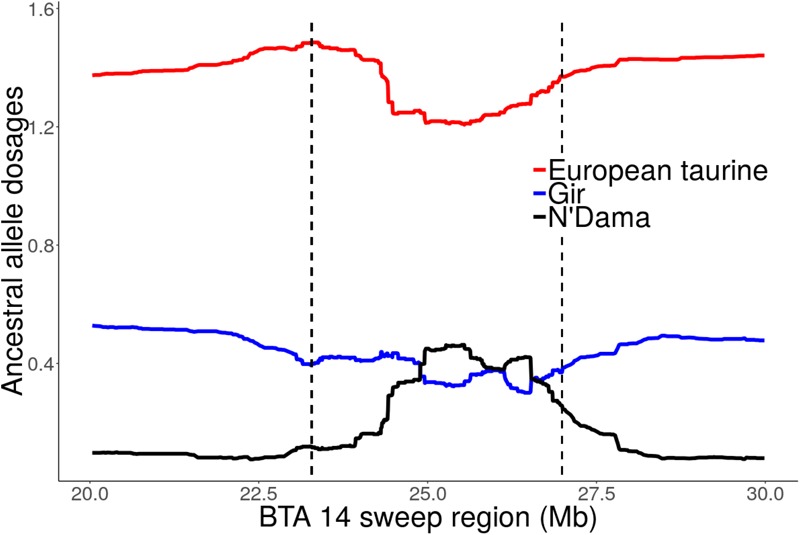
Local ancestral allele dosage on BTA 14 sweep region detected by iHS at 23.28 *–* 26.99 Mb for Tanzanian crossbred cattle. The dashed vertical lines delimit selective sweep region. The *y*-axis is the average ancestral allele dosage estimate for 324 unrelated individuals.

Focusing on the strong candidate selection region on BTA14 at 23.28 – 26.99 Mb, a quite striking observation is the excess AT ancestry dosage (>2 SD above the mean) and a corresponding decline in European taurine ancestry (Figure 8 and Supplementary Figure S7). The highest African taurine dosage (23%) corresponded to position 25505663 (Grubbs test for one outlier *G* = 2.93, *U* = 0.998, *p*-value = 1). Similarly, we observed elevated AT ancestry (>2 SD above the mean) on BTA 6 sweep region at 74.68 – 78.32 Mb (data not shown). When considering only the unrelated individuals from Rungwe population (*N* = 178) and the putative source populations (i.e., combined European taurine, EASZ and N’Dama), we observed a substantial increase in AT ancestry (>3 SD) on BTA 6 at ∼77 Mb (Supplementary Figure S7). The global ancestry for Rungwe population was estimated at 78, 7, and 15% for European taurine (ET), African taurine (AT), and indicine components, respectively. However, at position 77789716 (Grubbs test for one outlier *G* = 3.29, *U* = 0.998, *p*-value = 1), we detected the highest African taurine allele dosage (39%) as shown in Supplementary Figure S8.

Given that the EASZ which is the major base population used for crossbreeding in East Africa is a stable admixed breed consisting of African taurine and Asian indicine ancestral background (Mbole-Kariuki et al., 2014), we needed to be sure of the source of the ancestral alleles being detected in high dosage. Thus, we ran ELI on EASZ BTA 14 data using Gir (Asian indicine breed) and N’Dama (African taurine) as putative ancestral populations while specifying 500 admixed generations similar to Bahbahani et al. (2017). The results confirmed excess AT ancestry (> 2 SD above the mean) in several genomic regions across the chromosome including the major sweep region detected on BTA 14 (Supplementary Figure S9).

## Discussion

Dairy farmers in the tropics face many challenges including disease pressure, poor feed availability, high temperatures and generally inappropriate management strategies. Understanding the genetic basis for adaptation and production in this environment is critical if productivity is to be maximized. Characterizing the genetic structure of the population under study is important to the evaluation of the location of specific differentiation and effect of management practices and the production system on the gene pool.

### Genetic Diversity and Structure

Based on heterozygosity measures, we found low genetic diversity in indicine (EASZ and Gir) and African taurine (N’Dama) breeds compared to European taurine (ET) breeds. This is possibly due to the fact that the GGP HD array is optimized for use in *Bos taurus* breeds and has a very low representation of indicine breeds (especially those of African origin) as did the bovine 50K SNP chip (Bovine HapMap Consortium, 2009). This ascertainment bias is reflected in the disproportionate distribution of MAF among the subpopulations, such that indicine and African breeds had lower diversity measures. The relatively high proportion of SNPs with high MAF in the Tanzanian crossbred cattle can be attributed to the fact that these cows are recent crossbreeds with a significantly high inheritance of European taurine ancestry. This high genetic variability presents an opportunity for implementation of genetic improvement programs targeting traits important in adaptation to local production environments, which are constantly changing due to continuous environmental perturbations (Thornton, 2010). The relatively low heterozygosity estimates for indicine and African taurine breeds observed in this study is in line with the distribution of MAF described earlier and is likely due to poor representation of SNPs originating from African cattle.

We observed differences in inbreeding coefficient estimates between European taurine and indicine breeds. Additionally, there was a trend for Lushoto cattle to have higher inbreeding estimates compared to Rungwe cattle. During field-work, it was noted that farmers in Lushoto had limited access to breeding options compared to those in Rungwe, such that they mostly had access to bulls for breeding as opposed to artificial insemination. Since the available dairy bulls were limited in number and distribution, the inbreeding estimates in these animals would be higher.

To accurately assess population structure, we utilized PCA-AiR for PCA analysis. Unlike other standard PCA approaches, PCA-AiR uses estimates of kinship coefficients to accurately capture population structure, even in the presence of admixture (Conomos et al., 2015). The clustering of Tanzanian cattle population as depicted by PCA plot suggest that they are not only highly admixed but also mainly crosses of Friesian and the East African Zebu. The dominance of Holstein and Friesian genetic components in East African crossbred cattle has been reported in previous study (Kim and Rothschild, 2014). The dispersion pattern observed in this study is similar to that reported for Kenya and Uganda crossbred cattle (Weerasinghe et al., 2013) and generally reflects farmer’s efforts in upgrading animals to high exotic breed content in a bid to increase productivity. ADMIXTURE results obtained in this study are in concordance with the PCA results and demonstrate the narrow range of breed types used by farmers in the study sites. The dominance of Holstein and Friesian breeds over other cattle breeds suggests a preference for milk yield as the dominant trait of importance. However, it is not clear why there is almost a complete absence of smaller-bodied dairy breeds (Jersey, Ayrshire, and Guernsey) which have lower nutritional demands and higher production efficiency; characteristics that would make them more appropriate for smallholder production settings (Bebe et al., 2003). This reversal of breed preference likely reflects poor access to breed choices available to farmers or a mismatch of farmer aspirations and what is possible in their production environments. A scheme for appropriate sire selection that matches farmer production system ought to be instituted.

### Signatures of Selection and Identification of Candidate Genes

Our main goal for selection signature analysis was to detect regions that show preferential selection in the genome of Tanzanian crossbred dairy cows. To accomplish this, we used three different but complementary statistical methods: iHS and XP-EHH and pcadapt. Use of a combination of methods for selection sweep detection enables many different emerging patterns of selection to be identified, while also improving the robustness of the reliability and accuracy of the analyses (Qanbari and Simianer, 2014). The iHS and XP-EHH approaches used in our analysis have been successfully applied in multiple other studies to identify signatures of selection in admixed cattle (e.g., Kim and Rothschild, 2014; Bahbahani et al., 2015, 2017). The pcadapt approach is an outlier detection method based the on PCA which has been demonstrated to be robust to population admixture (Luu et al., 2017).

Failure to detect significant selection signals by XP-EHH approach is related to the FDR threshold used. For example, we found that minimum FDR threshold of 10% was required to capture comparable number of selection signals to that for iHS and pcadapt. Another reason could due to the heterogeneity of our admixed study sample. It is important to point out that we used a combined dataset of Rungwe and Lushoto cattle populations in all the analyses. This limits the power of XP-EHH since it is designed to detect differentiation of alleles among populations (Sabeti et al., 2007).

The putative selective sweeps regions that were detected by iHS and pcadapt have been widely reported in the literature. Nonetheless, distinguishing between true signatures of selection and those arising from natural phenomena such as admixture and genetic drift remains a challenging task (Akey et al., 2002). Additionally, SNP ascertainment bias toward European taurine alleles remains a major drawback to genetic analyses in crossbred populations (Lachance and Tishkoff, 2013), especially when considering populations comprising African breeds which tend to be under-represented in the majority of the SNP arrays used. Apart from that, false positives arising from selection signature analysis, continue to be a great concern and complicate the identification of true selection signatures (de Simoni Gouveia et al., 2014; Zhao et al., 2015). To date, little or limited efforts have been directed at estimating the magnitude of such bias on selection signature analyses. Thus, to limit possible false positives, we applied a stringent FDR threshold at 1% for all analyses. In addition, we adopted a SNP clustering approach in a bid to limit false positive detection associated with single-marker analyses. This clustering is akin to that of Johansson et al. (2010) where candidates SNPs used to qualify a selection sweep regions were required to be contiguous and not separated by >1 Mb for two divergent inbred chicken lines. However, our clustering was based on contiguous SNPs < 500 kb, considering previous studies indicating that LD in cattle does not exceed 500 kb (McKay et al., 2007).

It is not surprising that most of the selective sweep regions that were detected as significant at 1% FDR threshold, but excluded by the clustering criterion were obtained by pcadapt, which is based on single SNP analysis (Supplementary Table S2). Although it is possible that the regions excluded by our criteria may have arose by chance due to drift, it does not preclude the role of selection. For example, the GHR (growth hormone receptor) gene which has been widely reported to be under strong selection in cattle was excluded based on the criteria. The possible reason for this exclusion could be related in part to the sparse marker density in our analysis. Indeed, recent studies have demonstrated that using dense sequencing data improves detection and resolution of selection regions (Boichard et al., 2016). As noted by Teshima et al. (2006), and due to demographic effects, the fact that we applied stringent FDR at 1%, does not mean that false positives are completely eliminated. However, the application of this dual strategy gives us very high confidence that the areas identified as being under selection, as rightly so identified. The overlap of candidate regions identified in this study with those reported in previous work further supports the role of selection in the detected genomic regions.

A strong candidate sweep region was detected on BTA 14 at 23.28 – 26.69 Mb mapping to several well-known genes including PLAG1, CHCHD7, TOX, XKR4, TGS, TMEM68, and LYN which have been associated with pleiotropic effect on many traits in cattle including growth, milk characteristics as well as feed intake (Karim et al., 2011; Lindholm-Perry et al., 2012; Fink et al., 2017). Strong evidence of causal mutation BovineHD1400007259 (rs109815800), responsible for stature has been mapped to the intronic region of the PLAG1 gene (Karim et al., 2011; Bouwman et al., 2018). By examining allele frequency pattern (Supplementary Figure S4) we confirmed that this gene is segregating in the Tanzanian crossbred population. The allele for the causal SNP is almost fixed in Holstein and Friesian as previously reported (Bouwman et al., 2018) as well as in the African taurine breed (Supplementary Figure S4). The intermediate allele SNP frequencies in the Tanzanian crossbred cattle possibly suggest selection in favor of medium-sized animals represented by the EASZ. Moreover, the large haplotype diversity observed in the admixed populations perhaps explains the large variations in body sizes of dairy animals, in which characterize smallholder dairy systems.

XKR4 gene associated with feed intake and growth traits (Lindholm-Perry et al., 2012), meat and carcass (Bolormaa et al., 2011; Porto Neto et al., 2012) and reproductive traits (Fortes et al., 2012; Takada et al., 2018) has been repeatedly detected to be under strong selection in composite cattle (Bahbahani et al., 2015; Taye et al., 2017; Yurchenko et al., 2018). In this study, the most significant SNP under selection (BTB-01532239) is located at position 24437778 on BTA 14 (| iHS| = -6.84, *p*-value = 7.8 × 10^-22^), suggesting a strong sweep for the derived allele (Voight et al., 2006). This SNP is located close to BTB-01530836 at position 24573257 (| iHS| = -6.34, *p*-value = 2.2 × 10^-10^), which was reported to be significantly associated with subcutaneous rump fat thickness in indicine and taurine–indicine composite cattle (Porto Neto et al., 2012). Notably, the SNP was not detected by pcadapt as significant based on our criteria. The possible explanation for this could be that the SNP is segregating across all the breeds examined, thus limiting the detection power for differentiation-based methods such as the pcadapt. Further studies are required to pinpoint causal mutations for the SNPs mapping to the XKR4 gene.

Notable candidate genes identified on BTA 6 at around 37 Mb include HERC6, HERC5, PPM1K, NCAPG and LCORL and ABCG2 have been previously reported and are associated with multiple biological processes. For example, HERC6 and HERC5 genes belong to the HERC family of ubiquitin ligases which are linked with lactation persistency in cattle (Do et al., 2017). ABCG2 is strongly associated with milk yield and composition (Cohen-Zinder et al., 2005) while NCAPG and LCORL have been linked with growth traits in many species including cattle (Randhawa et al., 2016). A missense mutation in LCORL has been implicated as a causal mutation for stature in the sweep region (Bouwman et al., 2018). The strongest outlier SNP (BovineHD0600010756) linked to the sweep region bounding the LCORL gene was located at position 38874495 (pcadapt Chi2 = 41.19, *p*-value = 1.1 × 10^-9^). Another strong selection region on BTA 6 at 71 Mb mapped to a well-known KIT gene responsible for white spotting in Holstein cattle (Hayes et al., 2010; Randhawa et al., 2016).

The small overlap between candidate sweep regions is related to statistical approaches used. iHS detects selection signals of extended haplotype homozygosity (Voight et al., 2006) whereas pcadapt detects loci with large allele frequency differentiation among populations (Luu et al., 2017). The overlapping candidate region (Table 2) harbors several genes including LYPLA1 which has been reported to be associated with meat quality traits in Nelore cattle (Magalhães et al., 2016) as well as feed intake in cattle (Lindholm-Perry et al., 2012).

### Local Ancestry of the Sweep Regions

While admixed populations offer a unique opportunity in localizing selection signatures, the likely false positives arising due to post-admixture genetic drift remains a concern especially for recently admixed breeds such the Tanzanian crossbred cattle (Bhatia et al., 2014; Khayatzadeh et al., 2016; Pierron et al., 2018). However, the excess AT ancestry observed in the sweep regions despite significantly low average genome-wide AT ancestry provide strong evidence for the role of selection in the study population. Indeed, under the absence of selection, it is expected that ancestry estimates should be even across the genome (Pierron et al., 2018). Despite excess AT ancestry in the sweep regions, there is little evidence (*p* = 1) for outlier SNPs. This can be expected given few (∼10 – 15) admixture generations in the Tanzanian crossbred cattle which may have not allowed enough time for selection to generate significantly detectable levels of AT ancestry in the sweep region and across the genome. As noted by Jin et al. (2012), these results suggest that large sample size (>1000 individuals) would be required in future to distinguish deviation in ancestry estimates that arise from selection versus those resulting from genetic drift or sampling error.

The African taurine breeds generally have small body size (Rege, 1999) and possess unique adaptations to harsh climatic conditions and endemic diseases such as trypanosomiasis (Mattioli et al., 2000; Lemecha et al., 2006; Kim et al., 2017). The scarcity of feeds and sustained exposure to multiple disease agents such as viruses, fungi, parasites, bacteria, which are prevalent in smallholder livestock production conditions (Thumbi et al., 2013; Maleko et al., 2018) are likely to have favored selection for African taurine alleles to cope with such challenges. The observed elevated AT ancestry in the study population as well EASZ (Supplementary Figure S9) at the regions showing selection sweeps support the role of selection in favor of AT haplotypes which most likely occurred post-admixture. Given the results obtained here, further studies are required to understand the functional significance of African taurine haplotypes in the tropical adaptations.

## Conclusion

In this study, we have characterized population structure and demonstrated evidence of selection signatures in the Tanzanian crossbred cattle. Population structure analysis shows that the crossbred cows are mostly crossbreds of Holstein and Friesian cattle but with a wide variation of admixture. Selection signature analysis revealed several selection signals involved in multiple biological pathways related to production and adaptation. Local ancestry analysis revealed elevated African taurine ancestry dosage in the major candidate selective sweep regions. These results will complement previously reported findings and allow a better understanding of the genetic architecture of admixed cattle in tropical environments. This understanding is critical in order to maximize production through the use of animals better equipped to cope with stresses in tropical dairy production systems.

## Data Availability

The genotype data for the study population (Tanzanian crossbred cattle) is available at 10.6084/m9.figshare.7332845. Access to reference genotypes is available by making direct requests to the respective owners as indicated in the acknowledgments section of the article.

## Author Contributions

FM conceived, designed and obtained funding for the study. EC and FM analyzed the data. EC drafted the manuscript. RB, YZ, JA, RM, and FM contributed to revisions and edits of the manuscript. All authors read and approved the final manuscript.

## Conflict of Interest Statement

EC was an employee of USOMI LTD at the time of manuscript preparation. FM is currently an employee of USOMI LTD. The remaining authors declare that the research was conducted in the absence of any commercial or financial relationships that could be construed as a potential conflict of interest. The reviewer BS and handling Editor declared their shared affiliation.

## Footnotes

1 http://www.ensembl.org/biomart
