# Supplementary material for: Signatures of Selection in Admixed Dairy Cattle in Tanzania
Source: Front Genet. 2018 Dec 19;9:607. doi: 10.3389/fgene.2018.00607 (PMC6305962; doi:10.3389/fgene.2018.00607)
Supplement: Supplementary file 1 [file Table_1.PDF]

Supplementary material

Signatures of selection in admixed dairy cattle in Tanzania

Evans Kiptoo Cheruiyot*, Rawlynce Cheruiyot Bett, Joshua Oluoch Amimo, Yi Zhang, Raphael Mrode, Fidalis Denis Mujibi

*** Correspondence:** Corresponding Author: evanskip1@gamil.com

Supplementary Table S1 Distribution of SNP number per autosomal chromosome, mean length (Mb), mean length of adjacent SNPs and average LD for the markers set after quality control

BTA	Number of SNPs	BTA length (Mb)	Mean length (kb)	Mean LD (r^2)
1	6821	158.86	23.29	0.41
2	6026	136.77	22.70	0.41
3	5342	123.15	23.05	0.43
4	5184	120.63	23.27	0.39
5	5659	121.18	21.41	0.44
6	6334	122.51	19.34	0.45
7	5373	112.61	20.96	0.45
8	4792	113.63	23.71	0.40
9	4605	105.69	22.95	0.42
10	4512	104.25	23.11	0.40
11	4568	107.28	23.49	0.40
12	3913	91.13	23.29	0.41
13	3593	84.23	23.44	0.40
14	4532	84.63	18.67	0.49
15	3635	85.27	23.46	0.42
16	3472	81.67	23.52	0.41
17	3196	75.13	23.51	0.40
18	2807	66.00	23.51	0.40
19	2683	64.04	23.87	0.39
20	3505	71.96	20.53	0.45

21	3039	71.57	23.55	0.41
22	2636	61.37	23.28	0.39
23	2263	52.47	23.19	0.43
24	3161	62.64	19.82	0.45
25	1840	42.80	23.26	0.41
26	2242	51.68	23.05	0.40
27	1966	45.39	23.09	0.40
28	1982	46.22	23.32	0.38
29	2155	51.49	23.89	0.40
**Total**	**111,836**	**2516.25**	**22.67**	**0.41**
