# Supplementary material for: Signatures of Selection in Admixed Dairy Cattle in Tanzania
Source: Front Genet. 2018 Dec 19;9:607. doi: 10.3389/fgene.2018.00607 (PMC6305962; doi:10.3389/fgene.2018.00607)
Supplement: Supplementary file 2 [file Table_2.pdf]

Supplementary Table S2 Candidate selection genes in the Tanzanian crossbred cattle identified based on false discovery rate at 1% and the clustering criterion for iHS and pcadapt analyses. This criterion required candidate SNPs used to qualify a selection sweep region to be contiguous and not separated by > 500 kb.

BTA	Gene start	Gene end	Gene name	Gene type	Detecting analysis
6	4903635	4903741	RF00001	rRNA	iHS
6	37683713	37728536	HERC5	protein_coding	iHS
6	37736138	37793279	HERC6	protein_coding	iHS
6	37876470	37898522	PPM1K	protein_coding	iHS
6	37913110	38030583	ABCG2	protein_coding	iHS
6	37920071	37920177	RF00026	snRNA	iHS
6	38765969	38812051	NCAPG	protein_coding	pcadapt
6	38840894	38992112	LCORL	protein_coding	pcadapt
6	71604758	71604864	RF00026	snRNA	pcadapt
6	71796318	71917431	KIT	protein_coding	pcadapt
7	51185666	51213742	BRD8	protein_coding	pcadapt
7	51214237	51222637	KIF20A	protein_coding	pcadapt
7	51223146	51243230	CDC23	protein_coding	pcadapt
7	51240605	51240731	RF01225	snoRNA	pcadapt
7	51278946	51308203	GFRA3	protein_coding	pcadapt
7	51313474	51344138	CDC25C	protein_coding	pcadapt
7	51344517	51348748	SLBP2	protein_coding	pcadapt
7	51349485	51358857	FAM53C	protein_coding	pcadapt
7	51349701	51349770	bta-mir-2459	miRNA	pcadapt
7	51361891	51418615	KDM3B	protein_coding	pcadapt
7	51421165	51427158	REEP2	protein_coding	pcadapt
7	51438727	51442500	EGR1	protein_coding	pcadapt
7	51683087	51684304		processed_pseudogene	pcadapt
7	51688098	51880519	CTNNA1	protein_coding	pcadapt
7	51745434	51745547	RF00001	rRNA	pcadapt
7	51803645	51806667	LRRTM2	protein_coding	pcadapt
7	51888630	52144488	SIL1	protein_coding	pcadapt
7	51944664	51945231		protein_coding	pcadapt
7	52207256	52207396	RF00090	snoRNA	pcadapt
7	52209220	52209419	RF00090	snoRNA	pcadapt
7	52218128	52244915	MATR3	protein_coding	pcadapt
7	52252774	52270426	PAIP2	protein_coding	pcadapt
7	52272186	52284986	SLC23A1	protein_coding	pcadapt
7	52285863	52287943	MZB1	protein_coding	pcadapt
7	52290536	52293580	PROB1	protein_coding	pcadapt
7	52295318	52300482	SPATA24	protein_coding	pcadapt
7	52305244	52337268	DNAJC18	protein_coding	pcadapt
7	52346079	52354152	ECSCR	protein_coding	pcadapt
7	52355941	52357730	SMIM33	protein_coding	pcadapt
7	52361793	52368150	TMEM173	protein_coding	pcadapt
7	52414547	52457128	UBE2D2	protein_coding	pcadapt
7	52505097	52513059	CXXC5	protein_coding	pcadapt
14	23511301	23558678	ATP6V1H	protein_coding	pcadapt, iHS
14	23561303	23613020	RGS20	protein_coding	pcadapt, iHS

14	23620858	23637730	TCEA1	protein_coding	pcadapt, iHS
14	23651477	23668800	LYPLA1	protein_coding	pcadapt, iHS
14	23675383	23680928	MRPL15	protein_coding	pcadapt, iHS
14	23724626	23725771		protein_coding	pcadapt, iHS
14	23735755	23737474	POLR2K	protein_coding	pcadapt, iHS
14	26448145	26500189	NSMAF	protein_coding	pcadapt, iHS
14	26631190	26941726	TOX	protein_coding	pcadapt, iHS
14	24295567	24610955	XKR4	protein_coding	pcadapt, iHS
14	24711327	24747118	TMEM68	protein_coding	pcadapt, iHS
14	25492090	25492184	RF00026	snRNA	pcadapt, iHS
14	23373836	23395443	OPRK1	protein_coding	iHS
14	23884842	23886642	SOX17	protein_coding	iHS
14	23990193	23999338	RP1	protein_coding	iHS
14	24019278	24100855		protein_coding	iHS
14	24747219	24772713	TGS1	protein_coding	iHS
14	24847257	24920713	LYN	protein_coding	iHS
14	24955079	24956324	RPS20	protein_coding	iHS
14	24955769	24955835	RF01277	snoRNA	iHS
14	24970516	24970679	RF00003	snRNA	iHS
14	24975950	24976948	MOS	protein_coding	iHS
14	25007291	25009296	PLAG1	protein_coding	iHS
14	25052885	25058779	CHCHD7	protein_coding	iHS
14	25067486	25067823		protein_coding	iHS
14	25105062	25117554	SDR16C5	protein_coding	iHS
14	25153583	25179651	SDR16C6	protein_coding	iHS
14	25544907	25560879	IMPAD1	protein_coding	iHS
14	26115608	26116813	FAM110B	protein_coding	iHS
14	26180764	26180919		protein_coding	iHS
14	26271677	26304080	UBXN2B	protein_coding	iHS
14	26349329	26358692	CYP7A1	protein_coding	iHS
14	26380394	26380557	RF00003	snRNA	iHS
14	26412824	26447192	SDCBP	protein_coding	iHS
18	12952027	12966203	C18H16orf95	protein_coding	pcadapt
18	12974880	13009289	FBXO31	protein_coding	pcadapt
20	5656110	5727730	NSG2	protein_coding	iHS
20	28315521	28506629	PARP8	protein_coding	iHS
20	4449109	4452189	DUSP1	protein_coding	pcadapt
20	4505275	4618413	ERGIC1	protein_coding	pcadapt
26	20206332	20276715	CNNM1	protein_coding	iHS
26	20285687	20310044	GOT1	protein_coding	iHS
26	20371949	20372055	RF00026	snRNA	iHS
26	20400227	20403772	NKX2-3	protein_coding	iHS
26	20613538	20684065	ABCC2	protein_coding	iHS
26	20694707	20777487	DNMBP	protein_coding	iHS
26	20756089	20756231	RF00492	snoRNA	iHS
26	20756299	20756381	RF00283	snoRNA	iHS
26	20829327	20862859	CPN1	protein_coding	iHS
26	21056133	21108833	PKD2L1	protein_coding	iHS
26	21132751	21133969		protein_coding	iHS
26	21141592	21148318	SCD	protein_coding	iHS

26	21240570	21244552	WNT8B	protein_coding	iHS
26	21248525	21280208	SEC31B	protein_coding	iHS
26	21470763	21546730	PAX2	protein_coding	iHS
26	21566316	21566435	RF00156	snoRNA	iHS
26	21630816	21670831	SLF2	protein_coding	iHS
26	21677156	21690795	SEMA4G	protein_coding	iHS
26	21685254	21692758	MRPL43	protein_coding	iHS
26	21692833	21698205	TWNK	protein_coding	iHS
26	21703370	21710503	LZTS2	protein_coding	iHS
26	21711025	21712824		protein_coding	iHS
26	21713122	21730353		protein_coding	iHS
26	21731746	21738756	SFXN3	protein_coding	iHS
26	21760437	21762788	KAZALD1	protein_coding	iHS
26	21823912	21829144	TLX1	protein_coding	iHS
