# Supplementary material for: Signatures of Selection in Admixed Dairy Cattle in Tanzania
Source: Front Genet. 2018 Dec 19;9:607. doi: 10.3389/fgene.2018.00607 (PMC6305962; doi:10.3389/fgene.2018.00607)
Supplement: Supplementary file 3 [file Table_3.pdf]

Supplementary Table S3 Selection genes detected in the Tanzanian crossbred cattle at false discovery rate of 1% but were excluded as candidates based on the clustering criterion. This criterion required candidate SNPs used to qualify a selection sweep regions to be contiguous and not separated by > 500 kb.

CHR	Genes tart	Gene end	Gene name	Gene type	Detecting analysis
1	84324177	84325181		processed_pseudogene	iHS
1	84324970	84525526	MCF2L2	protein_coding	iHS
1	84366238	84428658	B3GNT5	protein_coding	iHS
2	111402467	111492915	SGPP2	protein_coding	pcadapt
2	111504465	111578408	FARSB	protein_coding	pcadapt
2	113274595	113287683	FAM124B	protein_coding	pcadapt
2	113372323	113487279	CUL3	protein_coding	pcadapt
2	120845481	120848463		protein_coding	pcadapt
2	120869240	120871886		protein_coding	pcadapt
2	120904601	120907738	ALPI	protein_coding	pcadapt
2	120910019	120913882		protein_coding	pcadapt
2	120925717	120933239	ECEL1	protein_coding	pcadapt
2	120975092	120982350	CHRND	protein_coding	pcadapt
2	120988439	120993968	CHRNG	protein_coding	pcadapt
2	120995644	121025344	EIF4E2	protein_coding	pcadapt
2	128710453	128783921	CLIC4	protein_coding	pcadapt
2	128848938	128877230	SRRM1	protein_coding	pcadapt
2	128916150	128930501	NCMAP	protein_coding	pcadapt
2	133224531	133246491	PLA2G5	protein_coding	pcadapt
2	133267318	133272112	PLA2G2A	protein_coding	pcadapt
2	133289295	133295334	PLA2G2A	protein_coding	pcadapt
2	133319863	133323729		protein_coding	pcadapt
2	133373263	133376607	PLA2G2E	protein_coding	pcadapt
2	133387211	133416030	OTUD3	protein_coding	pcadapt
3	538285	538916		protein_coding	pcadapt
3	553190	575832	GPR161	protein_coding	pcadapt
3	587034	768209	DCAF6	protein_coding	pcadapt
3	670582	670693	RF00201	snoRNA	pcadapt
3	94917406	94985342	RAB3B	protein_coding	pcadapt
3	95041110	95122769	NRDC	protein_coding	pcadapt
3	95045041	95045116	bta-mir-761	miRNA	pcadapt
3	95124031	95288435	OSBPL9	protein_coding	pcadapt
3	120904562	120960962	HDLBP	protein_coding	pcadapt
3	120968024	120988257	Sep-02	protein_coding	pcadapt
3	121053424	121065029		protein_coding	pcadapt
3	121065394	121074671	STK25	protein_coding	pcadapt
3	121098866	121111324	BOK	protein_coding	pcadapt
3	121118653	121153701	THAP4	protein_coding	pcadapt
4	10372349	10419397	HEPACAM2	protein_coding	pcadapt
4	10432174	10564199	VPS50	protein_coding	pcadapt
4	78221619	78492463	HECW1	protein_coding	pcadapt
5	35059697	35150452	ANO6	protein_coding	iHS
5	35174130	35174846		protein_coding	iHS
5	106812249	106852877	PRMT8	protein_coding	iHS
5	106975989	107038059	TSPAN11	protein_coding	iHS

5	19276348	19280028	DUSP6	protein_coding	pcadapt
5	56179509	56186416	B4GALNT1	protein_coding	pcadapt
5	56190025	56197400	PIP4K2C	protein_coding	pcadapt
5	56201577	56205117	DTX3	protein_coding	pcadapt
5	56205533	56212752	ARHGEF25	protein_coding	pcadapt
5	56214540	56219323	SLC26A10	protein_coding	pcadapt
5	56217962	56220534		protein_coding	pcadapt
5	56233023	56258780	KIF5A	protein_coding	pcadapt
5	56262302	56275883	DCTN2	protein_coding	pcadapt
5	56276101	56281781	MBD6	protein_coding	pcadapt
5	56285008	56289214	DDIT3	protein_coding	pcadapt
5	56289163	56307931	MARS	protein_coding	pcadapt
5	56293171	56293243	bta-mir-2430	miRNA	pcadapt
5	56313933	56321238	ARHGAP9	protein_coding	pcadapt
5	70323198	70395056	RFX4	protein_coding	pcadapt
5	70407271	70507072	RIC8B	protein_coding	pcadapt
6	4372937	4376146	NDNF	protein_coding	pcadapt
7	27820355	27998816	MEGF10	protein_coding	pcadapt
7	35984487	36107754	DMXL1	protein_coding	pcadapt
11	2730998	2762524	CNNM3	protein_coding	pcadapt
11	2768909	2773197	ANKRD23	protein_coding	pcadapt
11	2775465	2782703	ANKRD39	protein_coding	pcadapt
11	2784990	2793644	SEMA4C	protein_coding	pcadapt
11	2798822	2907015	FAM178B	protein_coding	pcadapt
11	2916017	2918064	COX5B	protein_coding	pcadapt
11	2929639	2943356	ACTR1B	protein_coding	pcadapt
11	62244354	62299938	UGP2	protein_coding	pcadapt
11	62301309	62377281	VPS54	protein_coding	pcadapt
11	78168945	78254093	LDAH	protein_coding	pcadapt
11	78270223	78274248	GDF7	protein_coding	pcadapt
11	78292606	78322321		protein_coding	pcadapt
11	96719400	96772227	PBX3	protein_coding	pcadapt
12	87918740	88313548	MYO16	protein_coding	pcadapt
14	67677676	67987801	STK3	protein_coding	pcadapt
18	22118201	22541532	FTO	protein_coding	pcadapt
18	22553058	22553182	RF00619	snRNA	pcadapt
18	22622803	22622916	RF00001	rRNA	pcadapt
19	40985698	41009183	MED24	protein_coding	pcadapt
19	40992603	40992706	RF01211	snoRNA	pcadapt
19	41017271	41042023	THRA	protein_coding	pcadapt
19	41040926	41048228	NR1D1	protein_coding	pcadapt
19	41069311	41079135	MSL1	protein_coding	pcadapt
19	41084252	41106062	CASC3	protein_coding	pcadapt
19	41109870	41122210	RAPGEFL1	protein_coding	pcadapt
19	41162358	41177893	WIPF2	protein_coding	pcadapt
19	41185975	41196948	CDC6	protein_coding	pcadapt
19	44509434	44529146	LSM12	protein_coding	pcadapt
19	44531725	44536439	G6PC3	protein_coding	pcadapt
19	44538303	44575554	HDAC5	protein_coding	pcadapt
19	44597496	44609787	C19H17orf53	protein_coding	pcadapt

19	44614052	44621587	ASB16	protein_coding	pcadapt
19	44629959	44633906	TMUB2	protein_coding	pcadapt
19	44633620	44641644	ATXN7L3	protein_coding	pcadapt
19	44648952	44660383	UBTF	protein_coding	pcadapt
19	44692189	44708381	SLC4A1	protein_coding	pcadapt
20	31793082	31834092	CCDC152	protein_coding	iHS
20	31890736	32199996	GHR	protein_coding	iHS
20	4641618	4671634	ATP6V0E1	protein_coding	pcadapt
20	4684744	4740893	CREBRF	protein_coding	pcadapt
20	4749695	4762879	BNIP1	protein_coding	pcadapt
20	4806231	4806349	RF00001	rRNA	pcadapt
20	4818457	4821534	NKX2-5	protein_coding	pcadapt
20	5409907	5410676		pseudogene	pcadapt
21	70827525	70839732	INF2	protein_coding	pcadapt
21	70845971	70861972	ADSSL1	protein_coding	pcadapt
21	70870027	70874415	SIVA1	protein_coding	pcadapt
21	70878138	70895537	AKT1	protein_coding	pcadapt
21	70903809	70905041	ZBTB42	protein_coding	pcadapt
21	70919570	70983506	CEP170B	protein_coding	pcadapt
26	46791127	47295654	DOCK1	protein_coding	pcadapt
26	47180463	47180538		miRNA	pcadapt
29	46407487	46417346	C29H11orf24	protein_coding	pcadapt
29	46456305	46456753		pseudogene	pcadapt
29	46485172	46573460	LRP5	protein_coding	pcadapt
29	46591196	46591315	RF00001	rRNA	pcadapt
