# Supplementary material for: Signatures of Selection in Admixed Dairy Cattle in Tanzania
Source: Front Genet. 2018 Dec 19;9:607. doi: 10.3389/fgene.2018.00607 (PMC6305962; doi:10.3389/fgene.2018.00607)
Supplement: Supplementary file 4 [file Image_1.PDF]

Supplementary Material

Signatures of selection in admixed dairy cattle in Tanzania

Evans Kiptoo Cheruiyot*, Rawlynce Cheruiyot Bett, Joshua Oluoch Amimo, Yi Zhang, Raphael Mrode, Fidalis D. N. Mujibi

***Correspondence:** Corresponding author: evanskip1@gmail.com

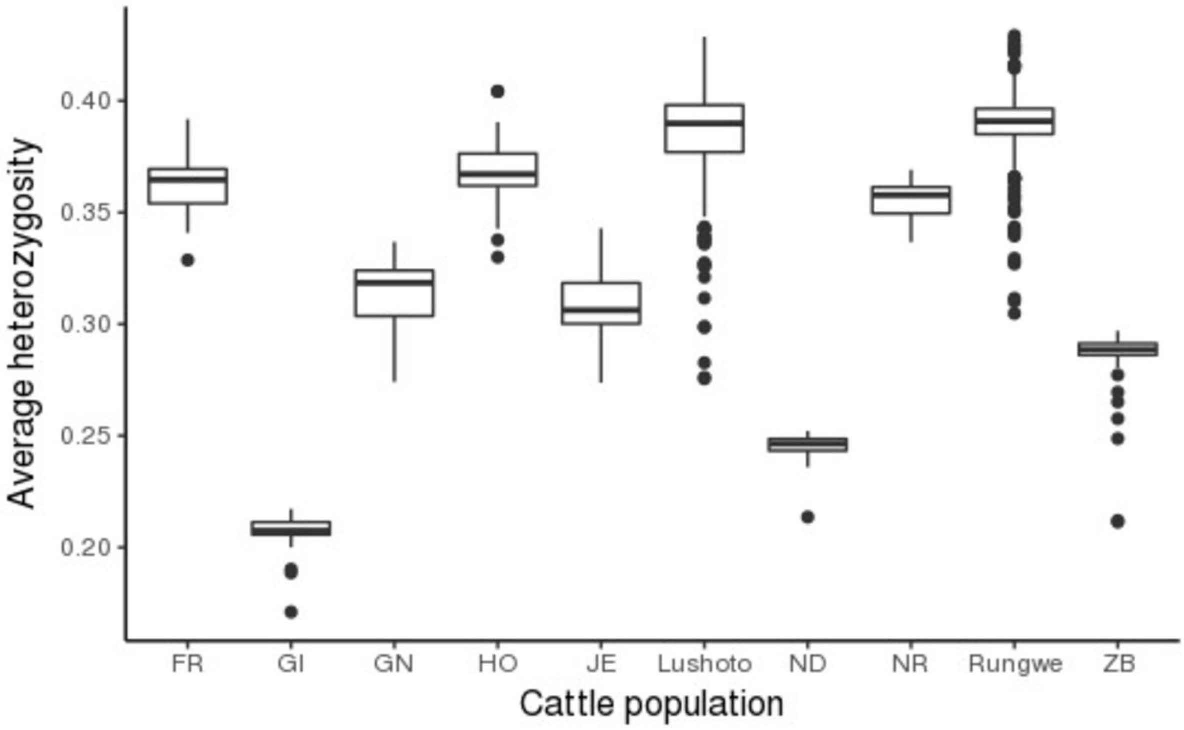

Supplementary Figure S1 Distribution of heterozygosity estimates in different groups of cattle. The breed groups are European taurines (Friesian (FR), Holstein (HO), Guernsey (GN), Jersey (JE), and Norwegian Red (NR)), African taurine (N'Dama (ND)) and Indicine breeds (Gir (GI), East African Shorthorn Zebu (ZB)). Lushoto and Rungwe represent Tanzanian crossbred cattle populations.

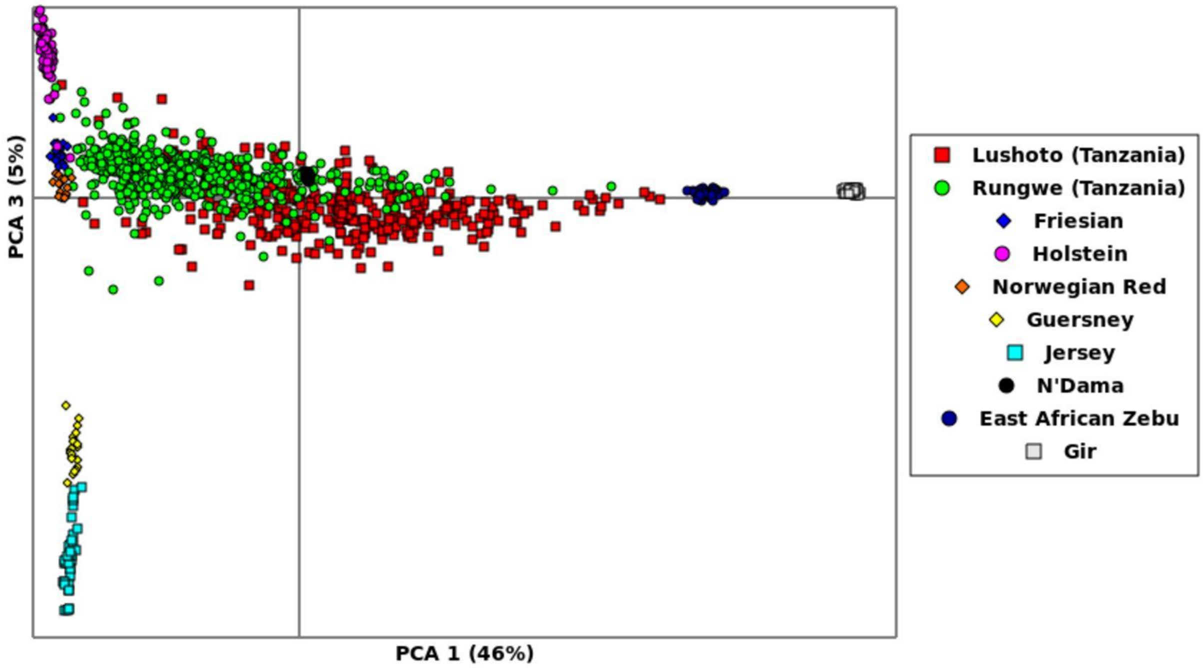

Supplementary Figure S2 Principal Component Analysis (PC) plot showing clustering of Tanzanian crossbred cattle (Rungwe and Lushoto) and reference breeds according to principal component 1 (PC1) and component 3 (PC3). Each point represents an individual animal colored as per the breed. The plot clearly shows the separation of Jersey and Guernsey from taurine breeds of Northern European origin.

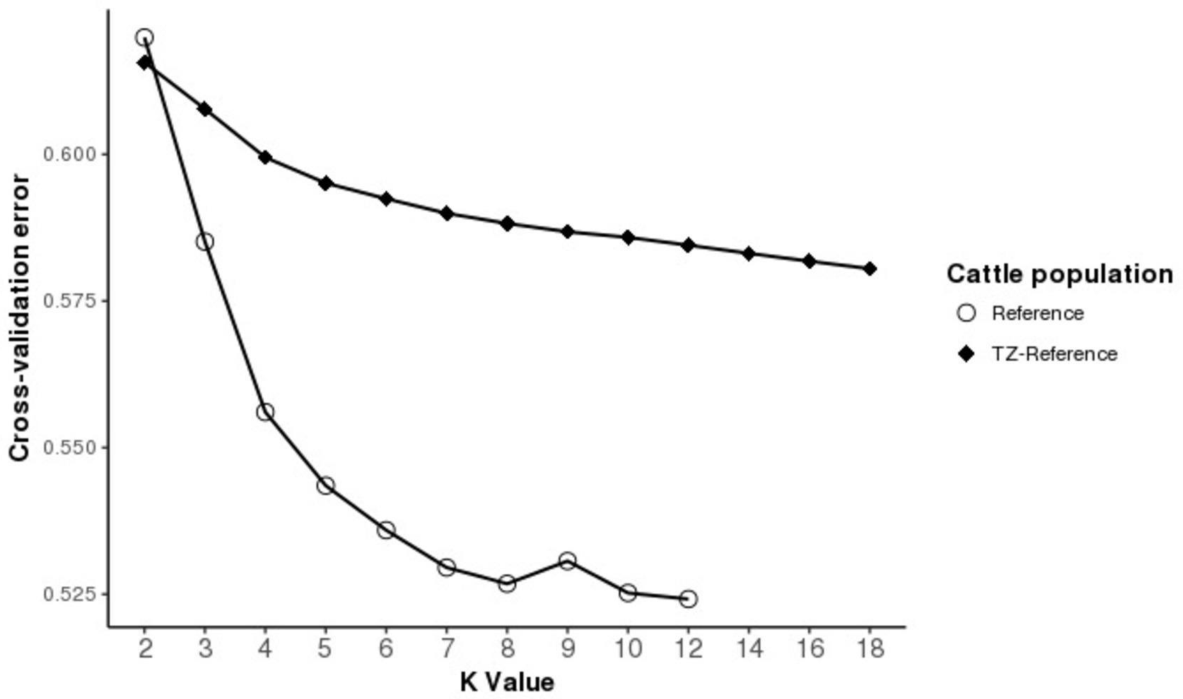

Supplementary Figure S3 Cross-validation error plot showing the best estimate for K (probable number of populations in the dataset) for a dataset with only reference breeds (Reference) and a combined dataset with both the Tanzanian crossbred cattle and reference breeds (TZ-Reference). The cross-validation errors for the reference breeds were plotted for K=2 to K=12 whereas for the combined dataset (TZ-Reference), it was plotted for K=2 to K=18. Eight reference breeds were included in the study: European taurine (Friesian, Guernsey, Holstein, Jersey, and Norwegian Red); Indicine (Gir and East African Shorthorn Zebu) and African taurine (N'Dama).

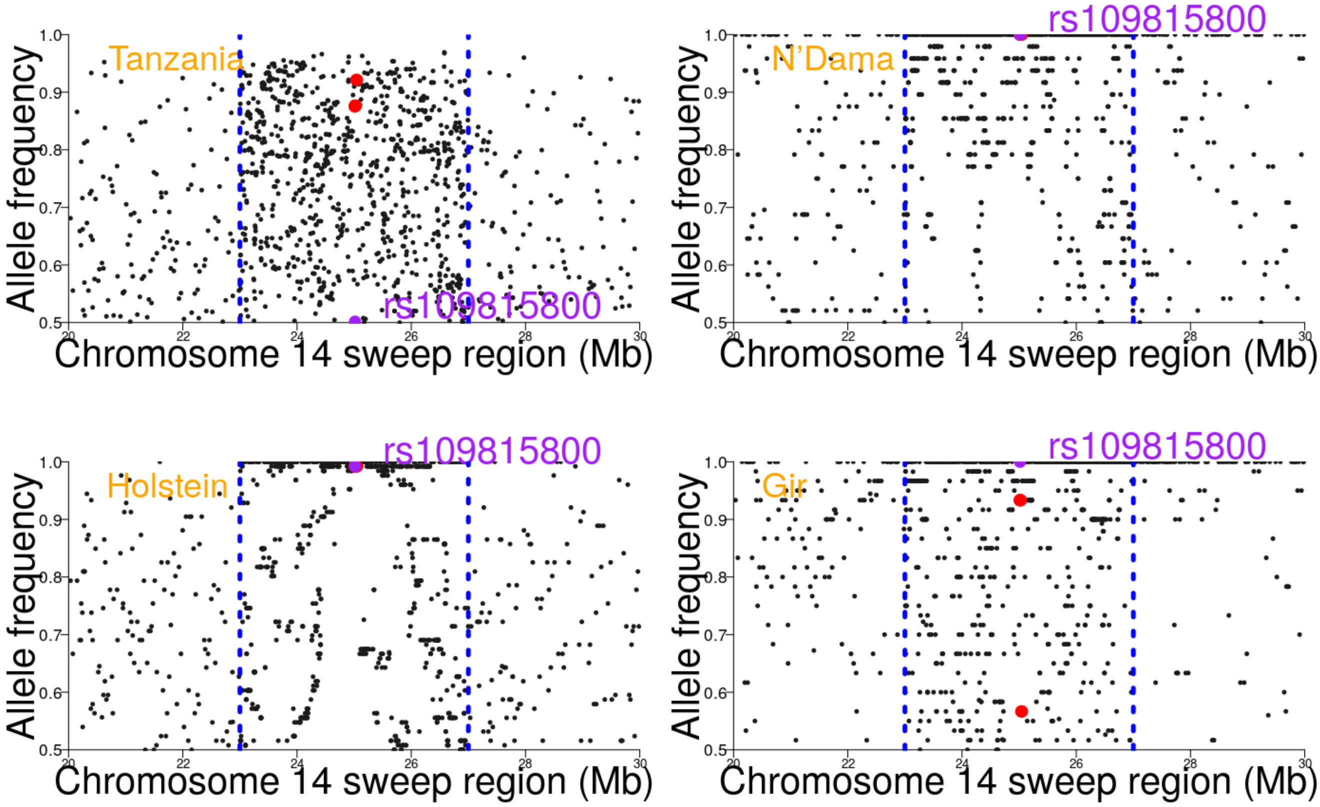

Supplementary Figure S4 Allele frequencies for the derived allele on the BTA 14 sweep region. The vertical dashed lines delimit the sweep region at 23.28 - 26.99 Mb. The purple colored SNP is potential causal mutation intronic to PLAG1 of Boitard et al. (2016) and Bouwman et al. (2018). The red colored SNPs are the other 7 SNPs at PLAG1 gene of Karim et al. (2011).

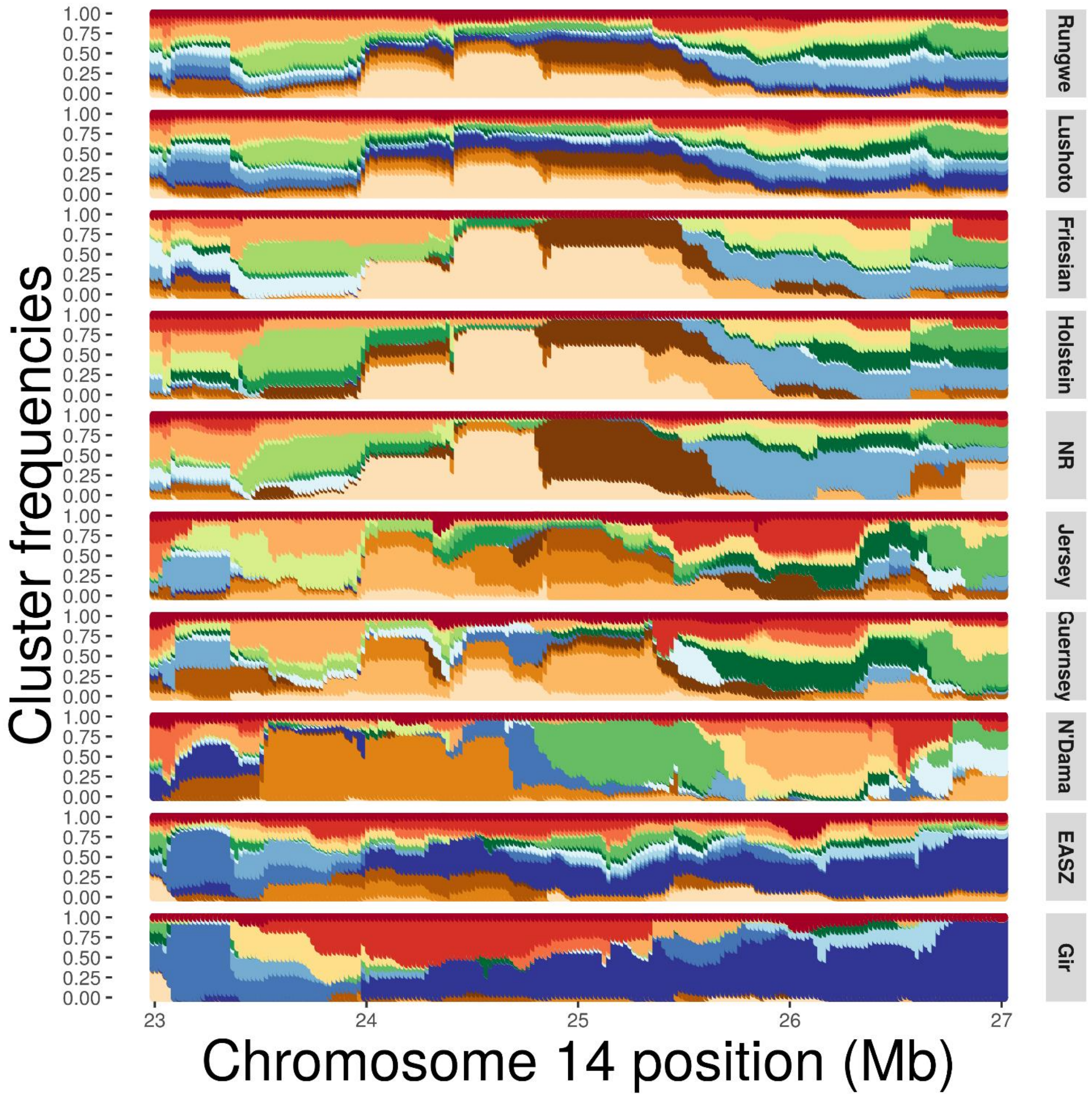

Supplementary Figure S5 Haplotype cluster frequencies on BTA 14 sweep region at 23.28 - 26.99 Mb. Haplotype clustering was performed based on Scheet and Stephens (2006) model into 20 clusters. The number of clusters were determined based on the cross-validation approach of Scheet and Stephens (2006).

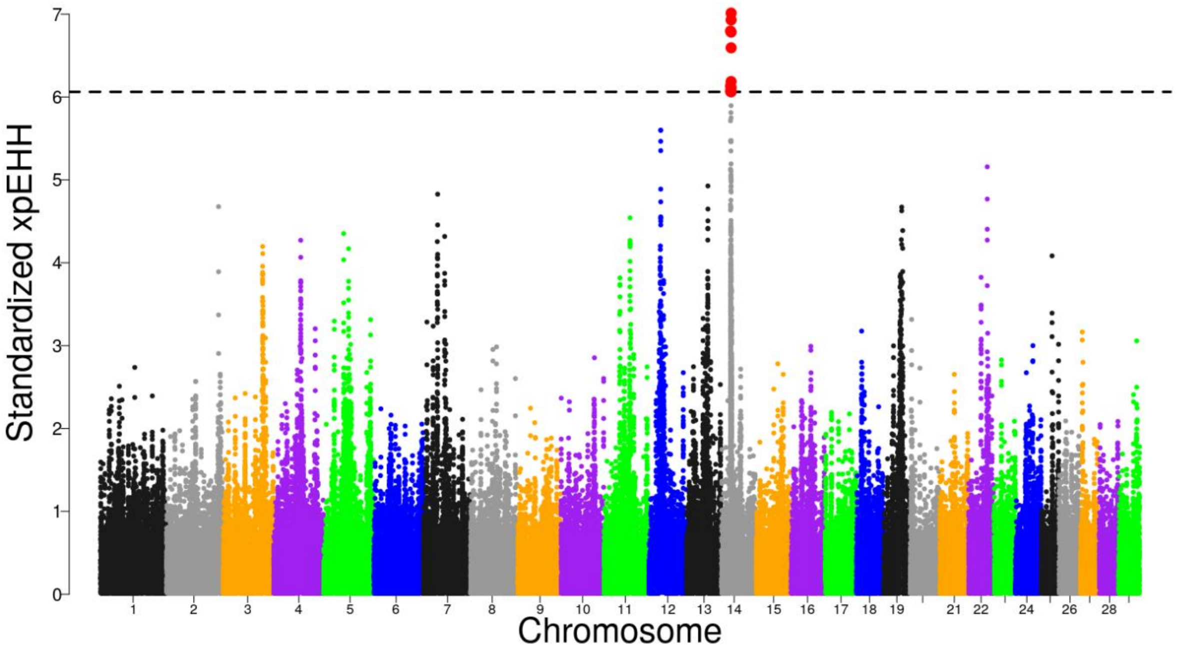

Supplementary Figure S6 Distribution of standardized XP-EHH scores in the Rungwe crossbred cattle versus the EASZ comparison. The dashed line corresponds to the false discovery rate (FDR) at 1% threshold.

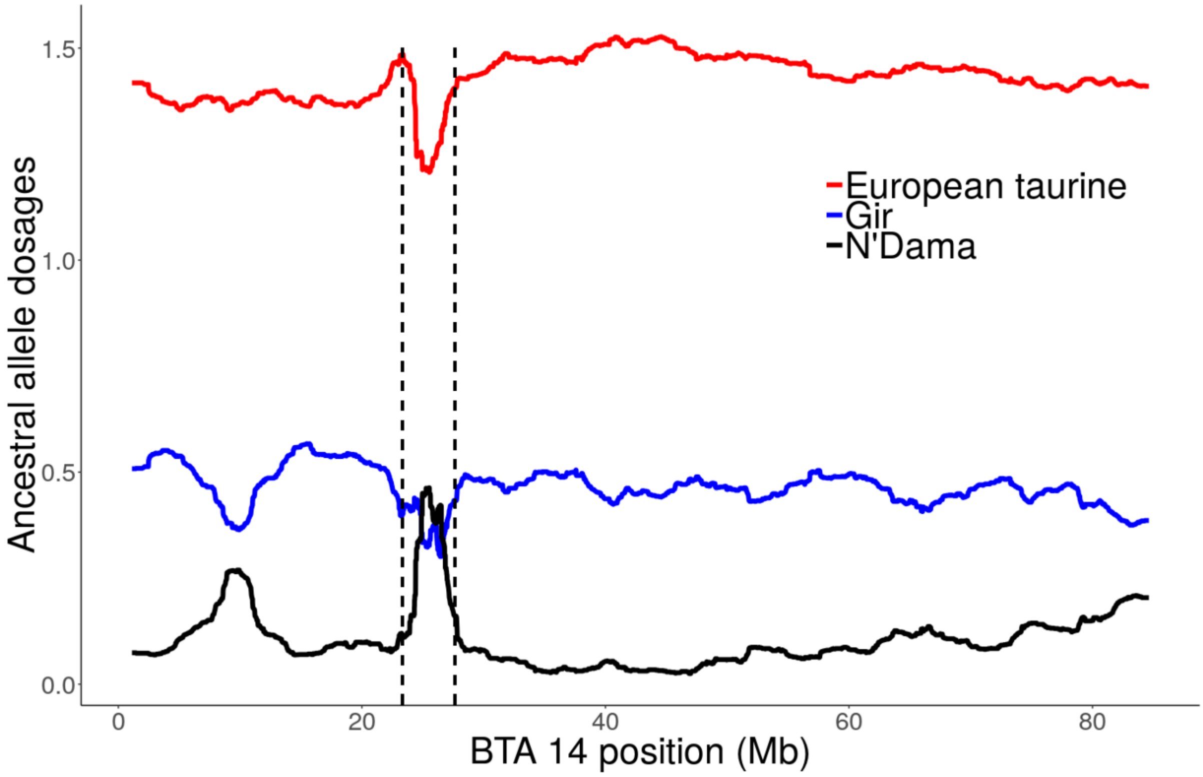

Supplementary Figure S7 Local ancestral allele dosage on BTA 14 for Tanzanian crossbred cattle. The dashed vertical lines delimit selective sweep region at 23.28 – 26.99 Mb. The y-axis is the average ancestral allele dosage estimate for 324 unrelated individuals.

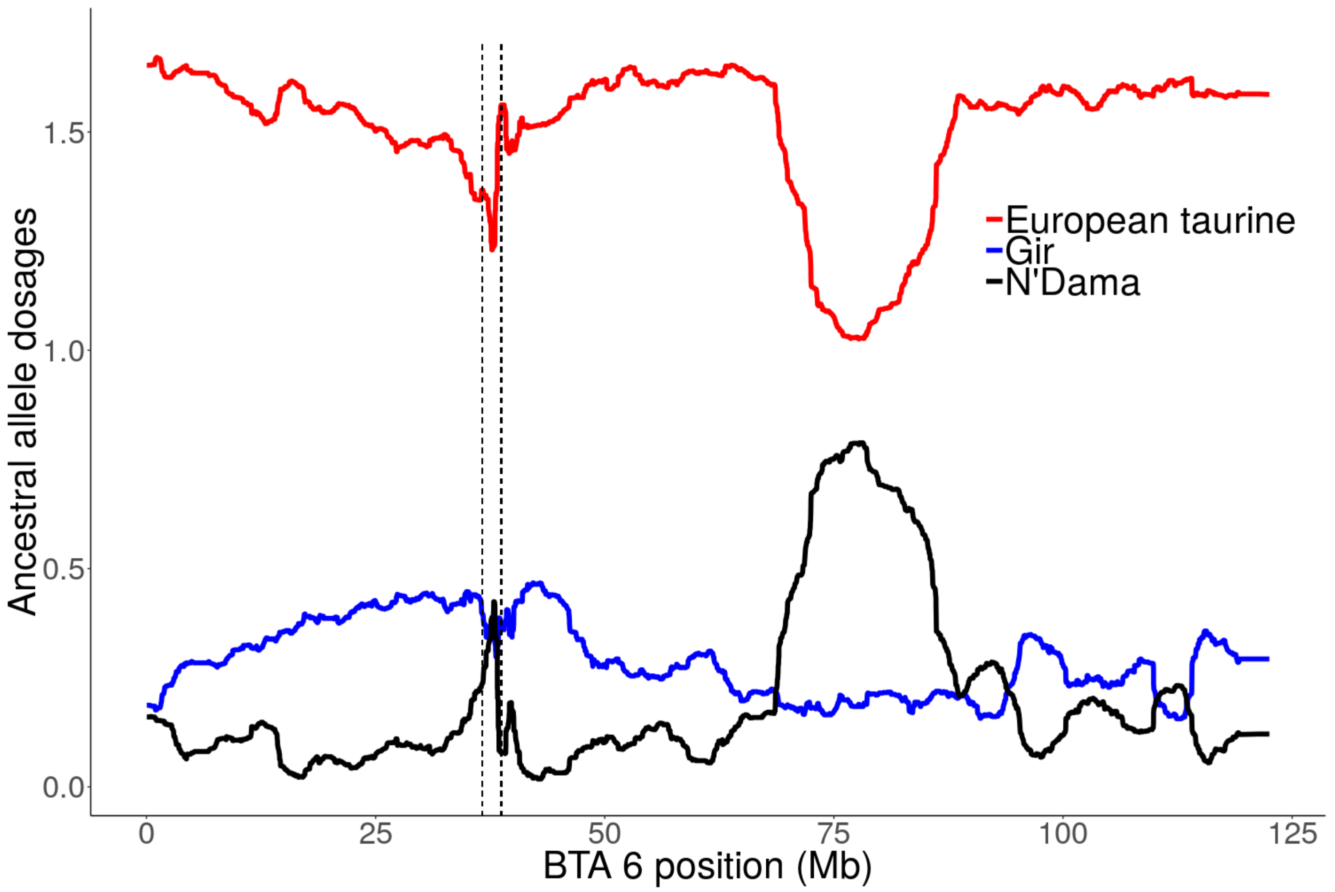

Supplementary Figure S8 Local ancestral allele dosage on BTA 6 for Rungwe cattle population. The dashed vertical lines delimit selective sweep region at 37.82 – 39 Mb. The y-axis is the average ancestral allele dosage estimate for 178 unrelated individuals.

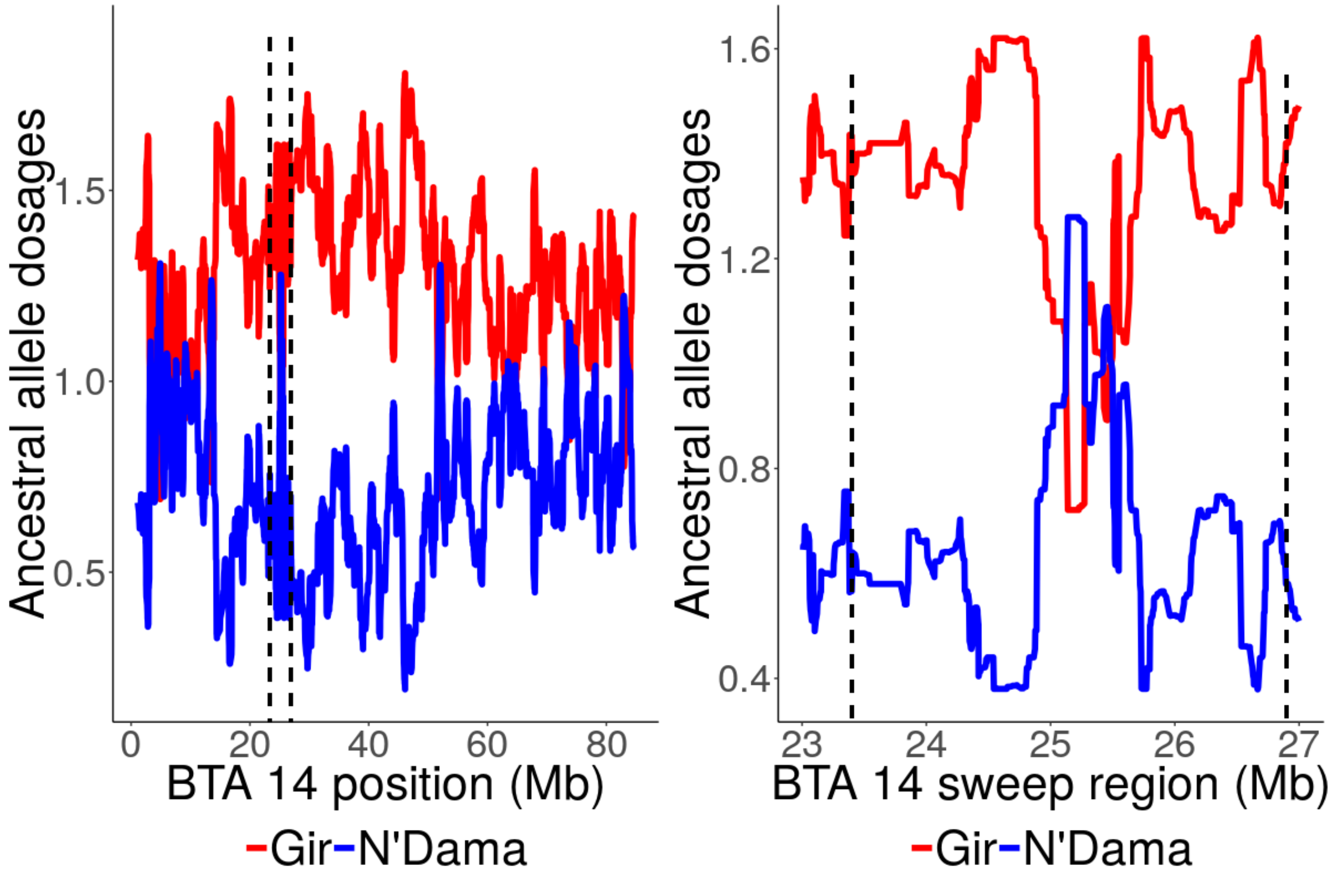

Supplementary Figure S9 Local ancestral allele dosage for East African Shorthorn Zebu (EASZ). The left and the right plot illustrates the distribution of the ancestral allele dosage across the whole chromosome and at the sweep region in the Tanzanian crossbred cattle, respectively. The dashed vertical lines correspond to the selective sweep region on BTA 14 at 23.28 – 26.99 Mb. The y-axis is the average ancestry estimates for 50 EASZ individuals.

References

Bouwman, A. C. et al. 2018. Meta-analysis of genome-wide association studies for cattle stature identifies common genes that regulate body size in mammals. Nature genetics 50: 362.

Karim, L. et al. 2011. Variants modulating the expression of a chromosome domain encompassing PLAG1 influence bovine stature. Nature genetics 43: 405.

Scheet, P., and M. Stephens. 2006. A fast and flexible statistical model for large-scale population genotype data: applications to inferring missing genotypes and haplotypic phase. The American Journal of Human Genetics 78: 629-644.
